# Schisandrin A Attenuates Diabetic Nephropathy via EGFR/AKT/GSK3β Signaling Pathway Based on Network Pharmacology and Experimental Validation

**DOI:** 10.3390/biology13080597

**Published:** 2024-08-08

**Authors:** Pengyu Wang, Qing Lan, Qi Huang, Ruyi Zhang, Shuo Zhang, Leiming Yang, Yan Song, Tong Wang, Guandi Ma, Xiufen Liu, Xiying Guo, Youzhi Zhang, Chao Liu

**Affiliations:** 1 School of Pharmacy, Hubei Engineering Research Center of Traditional Chinese Medicine of South Hubei Province, Xianning Medical College, Hubei University of Science and Technology, Xianning 437100, China; pengyuwang204@gmail.com (P.W.); 18950875093@163.com (Q.L.); hbkjhuangqi@163.com (Q.H.); zs751469566@163.com (S.Z.); 15055368534@163.com (L.Y.); 18771193739@163.com (Y.S.); wangtongjuly@163.com (T.W.); maguandi9905@163.com (G.M.); 2 School of Pharmacy, Hubei University of Chinese Medicine, Wuhan 430000, China; zhangry@hbust.edu.cn; 3 Hubei Key Laboratory of Diabetes and Angiopathy, Medical Research Institute, Xianning Medical College, Hubei University of Science and Technology, Xianning 437100, China; lxiufen2023@126.com (X.L.); gxyangela@163.com (X.G.); liuchao@hbust.edu.cn (C.L.); 4 School of Pharmacy, Xianning Medical College, Hubei University of Science and Technology, Xianning 437100, China

**Keywords:** Schisandrin A, diabetic nephropathy, EGFR, AKT/GSK-3β, network pharmacology, rat

## Abstract

**Simple Summary:**

End-stage renal disease is mainly caused by diabetic nephropathy, which has a complex pathogenesis and currently has no effective treatment. Schisandrin A has a wide range of pharmacological activities, including antioxidant, apoptosis inhibition, and immune regulation, but its pharmacological mechanism of action on diabetic nephropathy is still unclear. This study aimed to explore the pharmacological mechanism of Schisandrin A in the treatment of diabetic nephropathy using a network pharmacology approach. Through in vivo experiments, hub genes and related signaling pathways were verified based on the results of network analysis. The results showed that Schisandrin A had a protective effect on diabetic nephropathy, EGFR might be a potential therapeutic target, and AKT/GSK-3β might be involved in this process. This will provide a certain theoretical basis for the further clinical application of Schisandrin A in the treatment of diabetic nephropathy.

**Abstract:**

Diabetic nephropathy (DN) is one of the common complications of diabetes and the main cause of end-stage renal disease (ESRD) in clinical practice. Schisandrin A (Sch A) has multiple pharmacological activities, including inhibiting fibrosis, reducing apoptosis and oxidative stress, and regulating immunity, but its pharmacological mechanism for the treatment of DN is still unclear. In vivo, streptozotocin (STZ) and a high-fat diet were used to induce type 2 diabetic rats, and Sch A was administered for 4 weeks. At the same time, protein–protein interaction (PPI) networks were established to analyze the overlapping genes of DN and Sch A. Subsequently, the Kyoto Encyclopedia of Genes and Genomes (KEGG) and Gene Ontology (GO) analyses were performed to determine the hub pathway. In addition, molecular docking was used to preliminarily verify the affinity of hub proteins and Sch A. Further, H&E staining, Sirius red staining, immunohistochemistry, immunofluorescence, and western blot analysis were used to detect the location and expression of related proteins in DN. This study revealed the multi-target and multi-pathway characteristics of Sch A in the treatment of DN. First, Sch A could effectively improve glucose tolerance, reduce urine microprotein and urine creatinine levels, and alleviate renal pathological damage in DN rats. Second, EGFR was the hub gene screened in overlapping genes (43) of Sch A (100) and DN (2524). Finally, it was revealed that Sch A could inhibit the protein expression levels of EGFR and PTRF and reduced the expression of apoptosis-related proteins, and this effect was related to the modulation of the AKT/GSK-3β signaling pathway. In summary, Sch A has a protective effect in DN rats, EGFR may be a potential therapeutic target, throughout modulating AKT/GSK-3β pathway.

## 1. Introduction

Diabetic nephropathy (DN) is one of the most common and dangerous microvascular complications of type 2 diabetes. DN patients develop proteinuria due to the thickening of the glomerular basement membrane, which progresses to massive albuminuria and a low glomerular filtration rate, ultimately leading to end-stage renal disease (ESRD) [1,2]. Currently, there are few treatments for DN. Existing treatments focus on lowering blood pressure, controlling blood sugar, and regulating lipids to improve hypercoagulability, delay the occurrence and progression of proteinuria, and protect renal function [3,4]. However, traditional therapies that strictly control blood pressure and blood sugar have been proven to be unable to prevent DN from developing into ESRD or reduce DN mortality [5]. Therefore, there is an urgent need to discover preventive therapies that may prevent the progression of DN.

In recent years, the effects of traditional Chinese medicine treatments have gradually emerged [6,7]. These treatments have been shown to regulate blood sugar and lipid metabolism, reduce kidney damage, delay kidney disease, and avoid glomerular sclerosis and fibrosis [8]. Medicinal plants are considered safer and more effective than synthetic drugs in treating diabetic nephropathy [9]. Schisandra chinensis is a traditional Chinese medicine that contains a bioactive lignan called schisandrin A (Sch A) and is the “king of medicine” in Schisandra chinensis mixtures. According to traditional Chinese medicine theory, Schisandra chinensis can be used to treat liver and kidney yin deficiency or yang deficiency syndrome and has the effect of “tonifying the kidney and activating blood circulation” [10,11]. A large number of studies have confirmed that Sch A regulates multiple signaling pathways to exert a series of pharmacological effects, including phosphatidylinositol 3-kinase/protein kinase B (PI3K/AKT) [12], NOD-like receptor pyrin domain-related protein 3 (NLRP3) [13], nuclear factor E2-related factor 2 (Nrf2) [14], mitogen-activated protein kinase (MAPK) [15], and nuclear factor κB (NF-κB) [16]. In addition, Sch A has been shown to be essential for the management of DN in diabetic mice [13]. However, due to the multi-target of Sch A treatment, the specific mechanism of achieving the therapeutic effect of DN remains unclear. Therefore, it remains extremely difficult to elucidate the exact mechanism of action of Sch A in the treatment of DN.

Network pharmacology has been used to study and elucidate the pharmacological mechanisms of traditional Chinese medicine [17]. Through the application of network pharmacology, the potential mechanisms of Chinese herbal compound preparations or single ingredient have been deeply studied [6]. This has led to a shift in the traditional model of drug development from the current “single target” research strategy to a “multi-target” research strategy [18]. In addition, molecular docking can be used to confirm the interaction between active ingredients and hub therapeutic targets [19].

This study is mainly divided into two sections: network pharmacology analysis and experimental verification. Firstly, the targets related to Schisandrin A and diabetic nephropathy were obtained through multiple pharmacology databases, and the hub genes and pathways were determined through protein–protein interaction (PPI) analysis, Gene Ontology (GO) analysis, and Kyoto Encyclopedia of Genes and Genomes (KEGG) pathway analysis, and preliminarily verified by molecular docking. Secondly, through a variety of experimental methods, the effect of Schisandrin A in treating DN was verified by in vivo experiments. The purpose of this study is to explore the fundamental mechanism of Sch A in preventing and treating DN. It aims to provide a certain theoretical basis for the clinical application of Schisandrin A.

## 2. Materials and Methods

### 2.1. In Vivo Animal Experiments

#### 2.1.1. Materials

Schizandrin A (catalog number: S115189, Aladdin, Shanghai, China) and streptozotocin (catalog number: S110910, Aladdin, Shanghai, China) were purchased from Aladdin Technology Co., Ltd. (Shanghai, China). Anti-phospho-AKT (S473) (catalog number: #9271), anti-AKT (catalog number: #9272), anti-phospho-GSK-3β (Ser9) (catalog number: #9336), and anti-GSK-3β (catalog number: #9315) were purchased from Cell Signaling Technology (Danvers, MA, USA); anti-GAPDH (catalog number: A19056), anti-β-actin (catalog number: AC026), anti-PTRF (catalog number: A18331), anti-Bcl2 (catalog number: A18415), and anti-Bax (catalog number: A12009) were purchased from ABclonal Biological Technology Co., Ltd. (Wuhan, China); and anti-cleaved-caspase-3 (catalog number: WL01992), anti-phospho-EGFR (Tyr1172) (catalog number: WL03432), and anti-EGFR (catalog number: WL0682a) were purchased from Wanlei Biological Technology Co., Ltd. (Shanghai, China). All antibodies were diluted 1:1000 in primary antibody dilution buffers (MB9881, Meilunbio Biological Technology Co., Ltd., Dalian, China) for Western blot. Peroxidase-conjugated goat anti-rabbit IgG (catalog number: L3012, 1:10,000) and Peroxidase-conjugated goat anti-mouse IgG (catalog number: L3032, 1:10,000) were purchased from Signalway Antibody LLC Co., Ltd. (Greenbelt, MD, USA). SP Kit (Rabbit) was purchased from ZSGB-Bio Co., Ltd. (Beijing, China). Urine micro albumin assay kit (catalog number: E038-1-1) and creatinine assay kit (catalog number: C011-2-1) were purchased from Nanjing Jiancheng Biological Engineering Research Institute Co., Ltd. (Nanjing, China).

#### 2.1.2. Animals and Experimental Procedure

**Figure 1:**
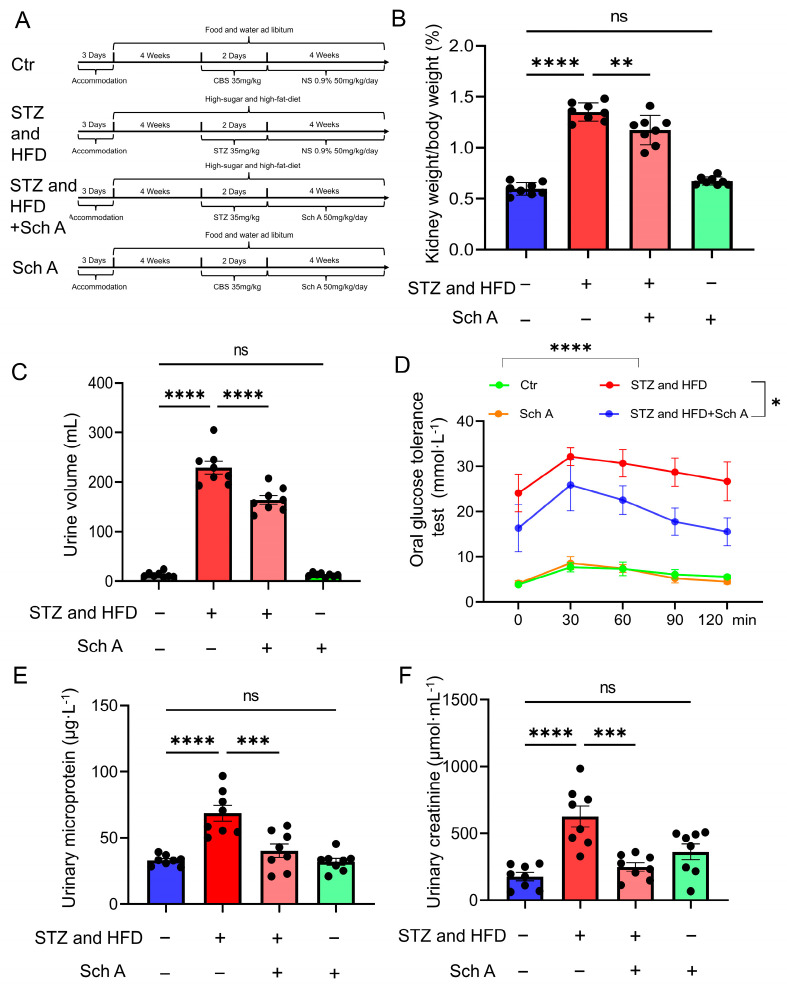
Effects of Sch A on rats with DN. (**A**) Timeline of rats modeling. (**B**) Total kidney weight/body weight. (**C**) 24 h urine volume. (**D**) Glucose tolerance. (**E**) Urinary microprotein. (**F**) Urinary creatinine in SD rats. *n* = 8. Color bars: Ctr (Blue), STZ and HFD (Red), STZ and HFD + Sch A (Pink); Sch A (Green). Abbreviations: CBS: citrate buffer solution; STZ: streptozotocin; NS: normal saline; Sch A: Schizandrin A; HFD: High-fat diet. The data are presented as mean ± standard deviation. Note: ns: no significant, * *p* < 0.05, ** *p* < 0.01, *** *p* < 0.001, **** *p* < 0.0001.

Forty mature male SD rats, weighing 200 ± 20 g with an age of 8 weeks, were provided from Beijing Weitonglihua Experimental Animal Technology Co., Ltd., Beijing, China. STZ in combination with a high-fat, high-sugar diet caused diabetes. The rats (2–5 per cage) were reared in a room where a steady ambient temperature of 22 ± 2 °C and humidity of 50 ± 5% were maintained in specific pathogen-free (SPF) conditions. The mice were exposed to a 12 h light–dark cycle. Following three days of adaptive feeding, the rats were split into two groups at random: the diabetic nephropathy group (STZ and HFD) and the control group (Ctr). The diabetic nephropathy group (*n* = 20) received a HFD (66.5% ordinary diet, 13.5% lard, and 20% sugar) whereas the control group (*n* = 20) received an ordinary diet. Following a 4-week period, the diabetic nephropathy group received intraperitoneal injections of STZ (dissolved in 0.1 M citrate buffer at pH 4.5) at a dose of 35 mg/kg/day for two consecutive days, whereas the control group received the same dosage of citrate buffer carrier. The rats’ fasting blood glucose (FBG) levels were measured 72 h after the STZ injection. If the rats’ FBG levels were ≥11.1 mmol/L, the induction of diabetes was deemed successful and used for additional experiments. The rats were divided into four groups: control (Ctr, *n* = 8); diabetic nephropathy (STZ and HFD, *n* = 8); diabetic nephropathy + Schizandrin A administration group (STZ and HFD + Sch A, *n* = 8) (50 mg/kg/day, i.g.,); control + Schizandrin A administration group (Ctr + Sch A, *n* = 8) (50 mg/kg/day, i.g.,). The Hubei University of Science and Technology’s Animal Care and Use Committee (IACUC Number: 2022-03-016) in Xianning, China, authorized all operations. The Declaration of Management of Laboratory Animals governing the Care and Use of Laboratory Animals was followed in the handling and care of the animals. Figure 1A shows our experiment’s comprehensive timetable.

#### 2.1.3. Oral Glucose Tolerance Test

All rats were fasted overnight (12 h) and intraperitoneally injected the next day with glucose solution (200 mg/mL) at a dose of 2 mg/g (10 mL/kg). Blood sugar was then measured at 0, 30, 60, 90, and 120 min after glucose injection.

#### 2.1.4. H&E Staining and Sirius Red Staining

H&E staining reveals the pathological structure of the kidney, and picrosirius red staining shows the degree of renal fibrosis [20]. According to the standard protocols [21], the kidney tissues of the rats were carefully removed after anesthesia using pentobarbital sodium and preserved in 4% paraformaldehyde for the whole night at 4 °C. After that, the tissues underwent gradual dehydration and were paraffin-embedded. After slicing the wax block into 4 μm pieces and spreading it out on the spreading table with clean water, it was dewaxed and rehydrated. Following the manufacturer’s directions, the rat kidney slices were stained using the H&E kit (catalog number: G1076, Servicebio, Wuhan, China). The steps for Sirius red staining are the same as for section dewaxing and rehydration. For eight minutes, immerse the slices in the ready-made Sirius red coloring solution (catalog number: G1056, Servicebio, Wuhan, China). Following their dehydration with anhydrous ethanol, the slices were immersed in n-butanol for ten minutes before being treated with xylene transparency. All images were obtained using the microscope (BX53; Olympus Corporation, Tokyo, Japan). 

#### 2.1.5. Immunohistochemistry Experiment and Immunofluorescence Staining

Relative protein expression levels (EGFR, PTRF, AKT, and GSK3β) are determined by immunohistochemistry. According to the standard protocols [22], to prepare kidney tissue sections, rat kidney tissues were perfused with ice-cold PBS and then perfused and fixed with ice-cold 4% paraformaldehyde. Next, they were dehydrated in a graded alcohol series and then embedded in paraffin. The wax-soaked tissues were cut into sections with a thickness of 4 μm (take a fixed piece every 40 μm). Finally, the sections were stored at room temperature. They were dewaxed in xylene and rehydrated by serially decreasing ethanol concentrations. Kidney sections were incubated in Tris-EDTA buffer at 100 °C for 20 min for antigen retrieval. Subsequently, the sections were permeabilized with 0.3% Triton X-100 and incubated with 10% normal goat serum for 1 h at room temperature to block nonspecific binding. And then, the diluted primary antibodies (EGFR, PTRF, AKT, and GSK3β, 1:100 dilution) were added in tissue slice. After that, color development was carried out using DAB and hematoxylin staining. Images were obtained using a microscope (BX53; Olympus Corporation, Japan). Finally, they were quantified by the IHC Profiler plugin in ImageJ (ImageJ bundled with 64-bit Java 8).

Similar to the preliminary experimental protocols of immunohistochemistry, tissue slices fixed in paraffin were deparaffinized, rehydrated, and then antigen repaired and endogenous peroxidase was eliminated. After sections were blocked, the sections were incubated with primary antibody (PTRF, 1:100 dilution) overnight at 4 °C and then with secondary antibody (goat anti-rabbit antibody conjugated to Alexa Fluor 488, 1:100 dilution) for 1.5 h at room temperature in the dark. Nuclei were counterstained with DAPI (C1002, 1:1000 dilution, Beyotime Biotechnology, Shanghai, China). Finally, antifade mounting medium was used to seal. Images were obtained using a fluorescence microscope (IX73; Olympus Corporation, Japan). Finally, they were quantified by ImageJ (ImageJ bundled with 64-bit Java 8).

#### 2.1.6. Protein Extraction and Western Blot

According to the standard protocols [23], the kidney tissues were immediately dissected, and homogenized using ice-cooled RIPA buffer that had been previously prepared. If necessary, a cocktail of protease and phosphatase inhibitors were added. The protein concentration was calculated by the BCA assays (20–30 μg). After denaturation, electrophoresis (60–120 V), and membrane transfer (75 min, 300 mA, PVDF membrane (Millipore, Billerica, MA, USA)), 5% skim milk was used at room temperature to prevent non-specific antibody binding. Specific primary antibodies were incubated with the membranes overnight at 4 °C, followed by washing three times with Tris-Buffered Saline Tween-20 (TBST). Membranes were then incubated with the corresponding secondary antibodies for 1 h at room temperature. Next, the image bands were exposed to the hypersensitive ECL luminous solution (MA0186, Meilunbio Biological Technology Co., Ltd., Dalian, China) using the multifunctional chemiluminescence imaging system (Bio-Rad GelDocTM XR and ChemiDocTM XRS System; Bio-Rad Laboratories, Inc., Hercules, CA, USA). Image J (version 1.53a; National Institutes of Health, Bethesda, MD, USA) was used to examine the protein bands and normalize them to GAPDH or β-actin.

#### 2.1.7. Statistical Analysis

The GraphPad Prism 9 (GraphPad Software, La Jolla, CA, USA) was used for all analyses. The findings are presented in the form of the mean ± standard deviation (SD). To obtain an average, each independent experiment was conducted at least three times. The Shapiro–Wilk test is employed to determine if the data follow the normal distribution. Bartlett’s and Brown Forsyth’s tests are used to check if they correspond to the homogeneity of variance. Tukey’s post hoc test was conducted after one-way analysis of variance (ANOVA) to evaluate the differences between various groups, and every possible comparison between the study groups was considered. It was deemed statistically significant differences existed when *p* < 0.05.

### 2.2. Network Pharmacology Analysis

#### 2.2.1. Acquisition of Overlapping Genes of Sch A and DN-Related Genes

The SwissTargetPrediction database (http://www.swisstargetprediction.ch (accessed on 28 February 2023)) was utilized to predict the targets of Sch A. The prediction results were exported as a file once the SMILES number of Sch A was entered and “Homo sapiens” was chosen.

To find the related therapeutic genes for diabetic nephropathy, “diabetic nephropathy” was separately put into the TTD (http://db.idrblab.net/ttd/ (accessed on 28 February 2023)), GeneCards (https://www.genecards.org (accessed on 28 February 2023)), OMIM (http://www.omim.org (accessed on 28 February 2023)), and DisGeNET databases (https://www.disgenet.org (accessed on 28 February 2023)). The targets were acquired through the Genecards database and were filtered based on the “Relevance score” median. Meanwhile, the genes acquired through the remaining three databases were kept. Following the above-mentioned disease genes collection and the removal of repeated data, the pertinent DN therapeutic targets were identified.

Subsequently, the overlapping genes between the disease targets and the Sch A targets were found and made a visible diagram in the Venn online platform. 

#### 2.2.2. Protein–Protein Interaction Analysis of Overlapping Genes

The overlapping genes obtained above were uploaded to the String database (https://cn.string-db.org/ (accessed on 1 March 2023)), the species was limited to “Homo Sapiens”, and the minimum confidence was set to 0.4. At the same time, the free nodes were hidden to construct a PPI network and visualized.

#### 2.2.3. Screening of the Hub Genes

The overlapping genes were loaded into the DIVID database, where they underwent KEGG enrichment analysis and GO enrichment analysis after selecting “OFFICIAL-GENE-SYMBLE”, setting the species to “Homo Sapiens”, and clicking “Gene List”. Lastly, the SRPLOT online (https://www.bioinformatics.com.cn/ (accessed on 1 March 2023)) mapping tool provided a visual representation of the analysis results.

The association network of Schizandrin A-target-pathway-diabetic nephropathy was built using Cytoscape 3.9.0 software. It was then analyzed using the network analyzer tool in Cytoscape software (version 3.9.0, National Institutes of Health, Bethesda, MD, USA). The hub genes were filtered based on degree value after the analysis results was exported.

#### 2.2.4. Molecular Docking and GRAMM Analysis

Molecular docking was used to preliminarily verify the affinity of Sch A to the hub genes. The 3D chemical structure of Sch A was acquired from the PubChem database (which could be translated to pdb format by OpenBabel-2.4.1), and the 3D crystal structures of the top three hub genes were retrieved from Uniprot database and stored in pdb format. To eliminate water, hydrogenate, configure ligands and receptors, and save the results as pdbqt files, AutodockTools-1.5.7 was utilized. Next, the docking box was configured, the GPF format exported, Autogrid-4 launched, the docking parameters and computation technique were chosen, Autodock-4 was launched to see the docking outcomes, and lastly, PYMOL 2.5 software was used to display the docking outcomes.

## 3. Results

### 3.1. Effect of Sch A on Rats with DN

The animal modeling timeline was shown in Figure 1A. Following modeling, they all had evident polydipsia, polyphagia, polyuria, and weight loss, along with symptoms such as villus clumping, diminution in self-cleaning activity, and listlessness. Meanwhile, the DN group had a much higher ratio of total kidney/body weight and a higher measure of urine volume (Figure 1B,C). In comparison with the DN group, the DN + Sch A group had a lower total kidney weight to body weight ratio and a lower urine volume. Additionally, Figure 1D demonstrated that rats receiving Sch A therapy had better glucose tolerance. Furthermore, compared to the DN group, the DN + Sch A group exhibited significantly lower urinary microprotein and urinary creatinine (Figure 1E,F). These findings showed that Sch A could attenuate kidney lesions.

**Figure 2:**
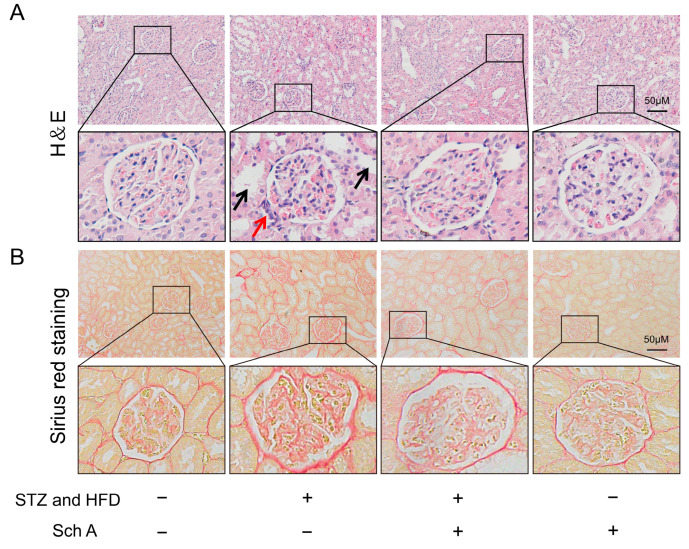
Sch A relieved morphological kidney injuries in diabetic rats. (**A**) Representative images of H&E staining in rat kidney tissue sections. The black arrows indicate inflammatory cell infiltration in the glomerular capillaries. The red arrows indicate the vacuolar degeneration of tubular epithelial cells and tubular dilation. Scale = 50 μm. (**B**) Representative images of Sirius scarlet staining in rat kidney tissue sections. Scale = 50 μm. Abbreviations: STZ: streptozotocin; Sch A: Schizandrin A; HFD: High-fat diet.

We also examined the histological alterations in the renal structure of rats with DN in order to investigate if Sch A had a protective impact on DN. In the DN group, tubular epithelial cells were tiny and vacuolated, and the glomerular capillaries were infiltrated by inflammatory cells. And the renal tubules also have a certain degree of ectasia, and congestion can be seen in the glomerular capillaries (Figure 2A). In the DN + Sch A group, granulocyte infiltration was also comparatively lower and the renal tubules were dilated. Additionally, we discovered that the glomeruli and tubule collagen fibers of the DN group had a more intense and noticeable red staining in Figure 2B. In addition, the red staining of glomeruli and renal tubules in the DN + Sch A group dramatically became shallow, the collagen distribution was homogenous, and the fibrotic nodules were greatly decreased compared with the DN group. These findings demonstrated that Sch A can clearly lessen renal impairment in DN rats.

### 3.2. GO and KEGG Enrichment Analysis of the Overlapping Genes

**Figure 3:**
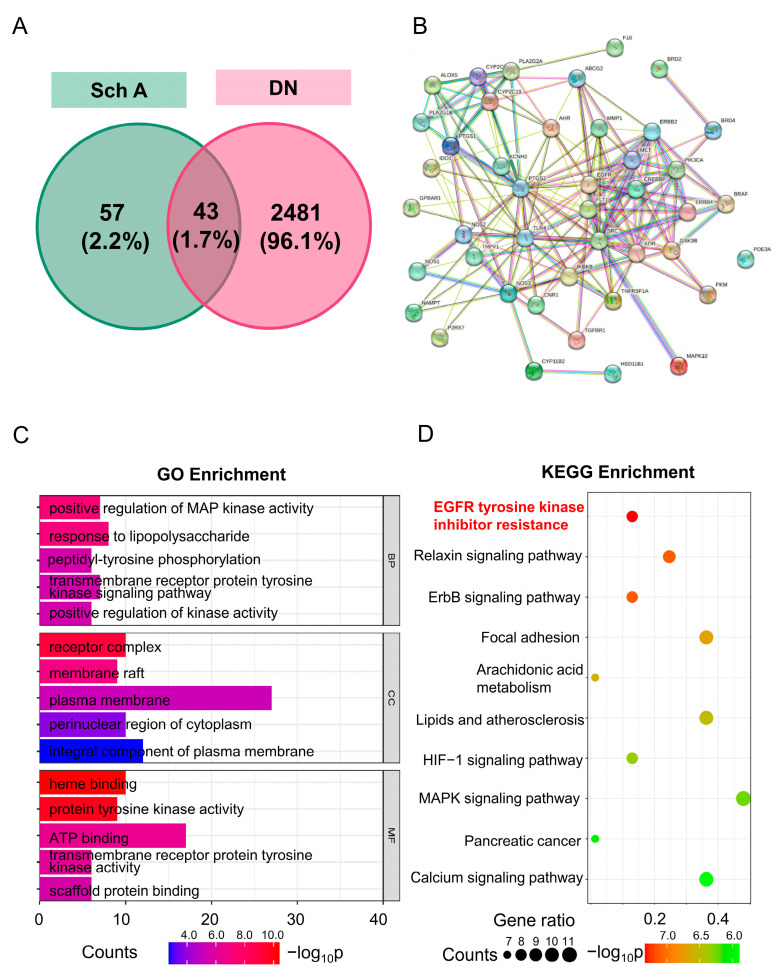
GO and KEGG enrichment analysis of intersection targets. (**A**) Venn diagram of overlapping genes of Sch A and DN therapeutic targets. (**B**) PPI network. (**C**) GO enrichment bar chart of the overlapping genes. (**D**) KEGG signaling pathway enrichment bubble diagram. Abbreviations: Sch A: Schizandrin A; PPI: Protein–Protein Interaction; GO: Gene Ontology; KEGG: Kyoto Encyclopedia of Genes and Genomes; BP: biological process, CC: cellular component; MF: molecular function.

A venn diagram was used to produce a total of 43 overlapping genes (Figure 3A). Following the analysis of PPI, 170 edges and 43 nodes, with an average node degree of 7.91, were obtained in this network (Figure 3B). The top 15 entities with the lowest *p* values for the biological process (BP), cellular component (CC), and molecular function (MF) of the GO enrichment analysis were displayed in Figure 3C. These findings suggested that Sch A may positively regulate MAP kinase activity to perform an ATP-binding role in components of the plasma membrane. Meanwhile, the top 10 KEGG pathways were performed in Figure 3D, indicating that the hub signaling pathway for Sch A in the treatment of DN may be EGFR tyrosine kinase inhibitor resistance.

### 3.3. The Hub Genes Were Obtained and Verified by Molecular Docking

**Figure 4:**
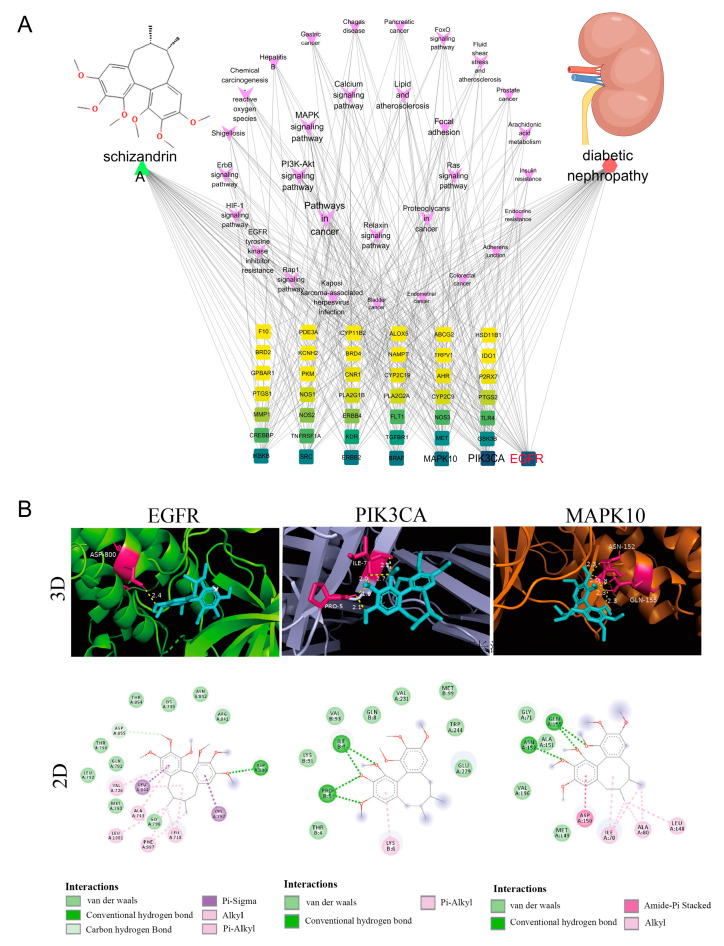
The hub proteins were obtained and verified by molecular docking. (**A**) Sch A-Target-Pathway-diabetic nephropathy network. The kidney was performed by Figdraw (https://www.figdraw.com/#/). (**B**) The molecular docking of Sch A with EGFR, PIK3CA, and MAPK10.

The association network diagram of Sch A-target-pathway-DN in Figure 4A was used to obtain the EGFR, PIK3CA, and MAPK10. Molecular docking was used to investigate the binding affinity of the hub proteins to Sch A in more detail (Figure 4B). Sch A exhibited a strong binding with EGFR (−7.18 kcal·mol^−1^), and the detail of the docking energy was shown in Table 1. Taken together, these results indicated that EGFR was most likely the hub genes that Sch A treated on DN.

### 3.4. Sch A Reduces the Expression Level of EGFR in Rats with DN

**Figure 5:**
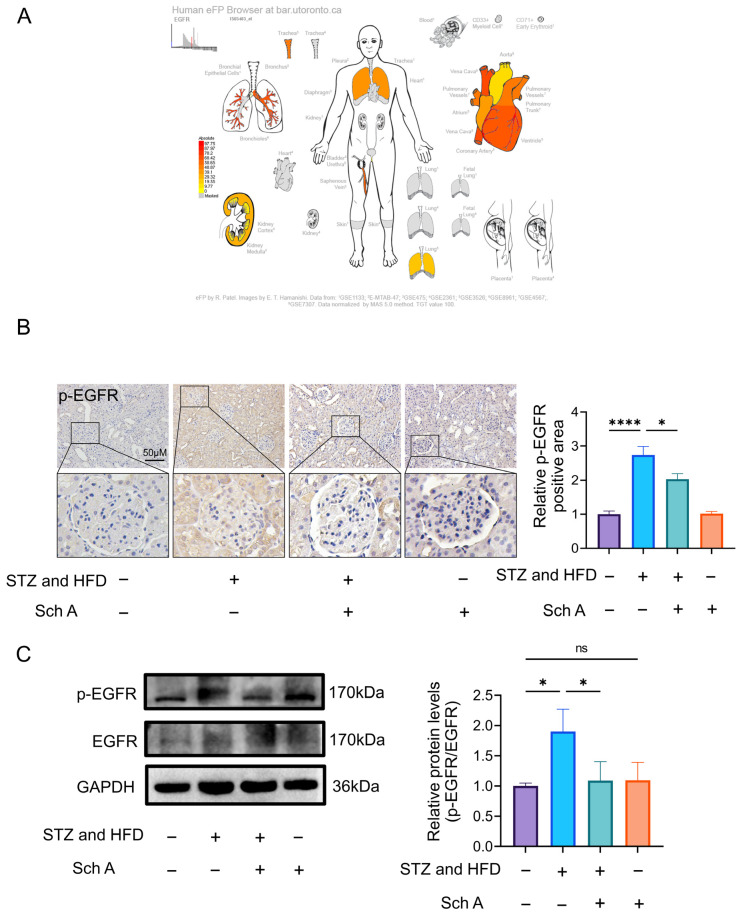
Sch A reduces the expression level of EGFR in rats with DN. (**A**) EGFR expression in skeletal immune digestive system. EGFR is highly expressed in the heart and widely expressed in the kidney. (**B**) The expression level of p-EGFR in the kidney was detected by immunohistochemistry. Scale = 50 μm; *n* = 5. (**C**) Western blot (Supplementary Materials) was used to detect the expression levels of EGFR and its phosphorylated protein, and the p-EGFR/EGFR ratio was calculated by statistical software; *n* = 3. The data were presented as mean ± standard deviation. Abbreviations: STZ: streptozotocin; Sch A: Schizandrin A; HFD: High-fat diet. Note: ns: no significant. * *p* < 0.05, **** *p* < 0.0001.

Numerous receptor tyrosine kinases (RTKs) have been linked to the pathophysiology of DN [24]. RTKs include the epidermal growth factor receptor (EGFR), which is a crucial component [25]. Following ligand binding, intracellular phosphorylation cascades initiate EGFR signaling. EGFR is abundantly expressed in glomeruli, proximal tubes, and collecting ducts [14]. In diabetic mice, EGFR phosphorylation is elevated, indicating that diabetic damage is mediated by its activation [26]. Furthermore, recent investigations have showed that pharmaceutical antagonists of EGFR efficiently prevent downstream cascade activation in diabetic mice. The histological expression of EGFR was detected to determine if it is a target of Sch A in the treatment of DN. As shown in Figure 5B, we discovered that EGFR expression was lower in the DN + Sch A group and higher in the DN group. Additionally, Figure 5C demonstrated that the p-EGFR expression level in DN group was much greater than the Ctr group. Nevertheless, following Sch A therapy, there was a reduction in p-EGFR expression. These findings showed that Sch A therapy for DN rats may focus on EGFR and that Sch A treatment may also decrease EGFR expression in the DN rats.

### 3.5. Effect of Sch A on AKT/GSK3β Pathway and Apoptosis Factors in Rats with DN

**Figure 6:**
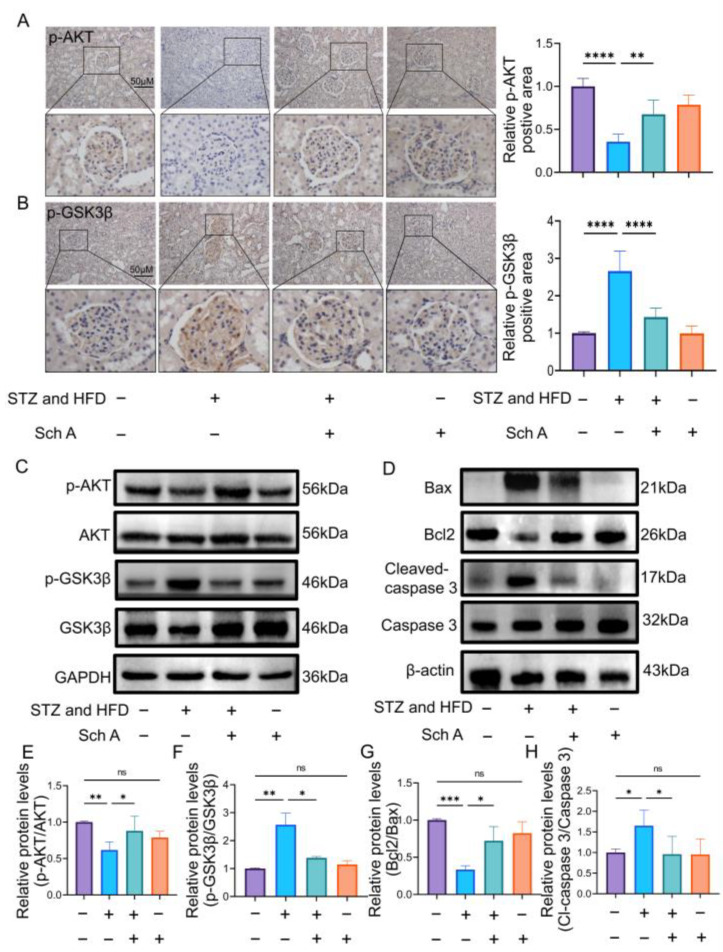
Effect of Sch A on AKT/GSK3β signal pathway and apoptosis factors in rats with DN. (**A**) Immunohistochemistry was used to detect p-AKT expression in the kidney (scale = 50 μm, *n* = 5). (**B**) Immunohistochemistry was used to detect p-GSK3β expression in the kidney (scale = 50 μm, *n* = 5). (**C**) The expression of AKT and GSK3β proteins in the kidney were detected by Western blot (Supplementary Materials). (**D**) The expression of Bax, Bcl2, and Cleaved-caspase3 proteins in the kidney was detected by Western blot (Supplementary Materials). (**E**,**F**) The quantitative evaluation of p-AKT/AKT and p-GSK3β/GSK3β; *n* = 4 or *n* = 3. (**G**,**H**) The quantitative evaluation of Bcl2/Bax and Cleaved-caspase3; *n* = 3 or *n* = 5. The data were presented as mean ± standard deviation. Abbreviations: STZ: streptozotocin; Sch A: Schizandrin A; HFD: High-fat diet; ns: no significant. Note: * *p* < 0.05, ** *p* < 0.01, *** *p* < 0.001, **** *p* < 0.0001.

The primary mechanism of Sch A in the treatment of DN is the EGFR tyrosine kinase inhibitor resistance pathway. Thus, we investigated further the alterations in GSK3β and AKT expression levels after the intervention of Sch A in DN rats, suggesting that the EGFR tyrosine kinase inhibitor resistance pathway was suppressed. As shown in Figure 6A,C,E, the DN group had a substantial decrease in AKT phosphorylation, but the DN + Sch A group had an increase in p-AKT protein production. Meanwhile, the kidneys in the DN group showed much higher levels of GSK3β phosphorylation, particularly in the glomeruli. Furthermore, the presence of Sch A resulted in a reduction in the expression of the p-GSK3β protein (Figure 6B,C,F). GSK-3β activation is linked to renal cell apoptosis, and accompanied by modifications in the kidney-induced downstream proteins Bax and Bcl2 [27]. As shown in Figure 6D,G,H, when DN was administered, Bcl2/Bax was dramatically reduced and cleaved caspase-3 was elevated in comparison to the Ctr group. However, Sch A clearly reversed these alterations. According to these findings, Sch A may suppress the EGFR tyrosine kinase, activate the expression of phosphorylate AKT, and therefore inhibit the phosphorylation of GSK3β.

### 3.6. The Relationship of PTRF with EGFR

**Figure 7:**
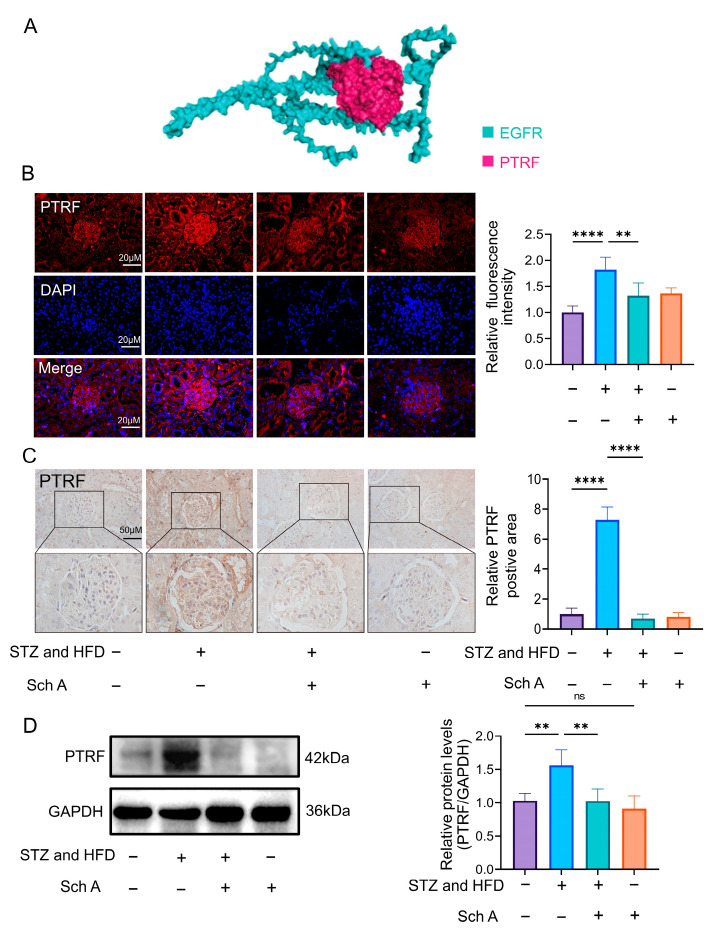
The relationship between PTRF and EGFR. (**A**) The interaction between EGFR and PTRF was obtained by activating GRAMM docking. Blue: EGFR, red: PTRF. (**B**,**C**) The expression of PTRF in renal tissues of diabetic nephropathy rats was detected by immunofluorescence (scale = 20 μm, *n* = 5) and immunohistochemistry (scale = 50 μm, *n* = 5). (**D**) Western blot (Supplementary Materials) was used to detect PTRF expression in the kidney; *n* = 5. The data were presented as mean ± standard deviation. Abbreviations: STZ: streptozotocin; Sch A: Schizandrin A; HFD: High-fat diet; ns: no significant. Note: ** *p* < 0.01, **** *p* < 0.0001.

Then, we discovered that polymerase I and transcript release factor (PTRF) and the EGFR of String and GRAMM exhibited a significant binding energy (interfacial area 1818.0A2, solvation free energy 27.9 kcal/mol) (Figure 7A). The EGFR/PI3K/AKT pathway controls PTRF expression, and increased PTRF expression causes diabetic wound healing to be delayed [28]. We examined PTRF expression to determine more about the connection between PTRF and EGFR. Initially, immunohistochemistry and Western blot demonstrated that the PTRF expression level matched the outcomes of immunofluorescence detection (Figure 7B–D). The findings demonstrated that the expression level of PTRF in the DN group was higher than that in the DN + Sch A group. Nevertheless, we also discovered that PTRF was mostly detected in glomeruli, peripheral capillaries, and vascular endothelium (Figure 7B,C). These results revealed that PTRF might bind to EGFR, and the reduced expression of PTRF may be related to the suppression of the EGFR tyrosine kinase by Sch A.

## 4. Discussion

Diabetic nephropathy is the most common cause of chronic kidney disease worldwide. Owing to the absence of therapeutic options, delaying the onset of DN has emerged as a difficult worldwide public health issue [29]. As we all know, traditional Chinese medicine has long been valued for its exceptional safety record and negligible adverse effects in clinical settings by Asians [30,31]. Schisandra chinensis is a drug that has been shown to successfully treat diabetic neuropathy in mice [11]. However, it is challenging to comprehend the precise pharmacological mechanisms behind traditional Chinese herbal medicine because the wide range of components used in it and the intricacy of the synergistic effects of prescriptions. The pharmacological activity of Sch A, a major monomer in Schisandrin chinensis fructus, has been demonstrated to have hepatoprotective, neuroprotective, anti-inflammatory, antiviral, antioxidative, and anticancer properties [32]. Thus, in order to methodically investigate the therapeutic mechanism of Sch A on DN, this study included network pharmacology, bioinformatics analysis, and experimental verification.

According to reported research, Sch A has prevented renal impairment in mice model of DN and shown a variety of pharmacological advantages [13]. However, further research is needed to fully understand the molecular process. In this study, the potential mechanism of Sch A was examined in diabetic rats as well as its potential to preserve the kidneys. Our findings demonstrated that Sch A successfully halted the progression of DN and reduced kidney damage. Additionally, it was shown that EGFR was a crucial regulator that facilitated the protective actions of Sch A in DN rats, with decreased EGFR expression. Furthermore, the expression of p-AKT was increased and p-GSK3β was decreased following Sch A therapy. All results considered, our research suggested that EGFR could be a potential treatment target for Sch A, eventually reducing renal damage in the diabetic rats by controlling the AKT/GSKβ pathway.

A total of one hundred putative genes matching to Sch A were retrieved using the SwissTargetPrediction database. Next, utilizing network pharmacology and bioinformatics techniques, this study investigated a prediction for targets of DN. From the disease databases, which include TTD, GeneCards, OMIM, and DisGeNET databases, DN-associated genes (2524) were gathered. These forty-three overlapping genes were thought to be crucial targets for Sch A to enhance DN. After that, we examined the therapeutic targets using GO and KEGG enrichment analysis. Therefore, Sch A might operate via the previously specified pathways. For instance, the formation and progression of DN are linked to inflammation, and several studies indicate that the MAPK signaling pathway is crucial for both the activation of inflammation and the cell damage brought on by excessive hyperglycemia [33]. The kidneys can be safeguarded and the DN inflammatory response can be decreased by blocking the MAPK signaling pathway [34,35]. The EGFR tyrosine kinase inhibitor resistance pathway was mostly linked to the therapeutic targets of Sch A against DN (Figure 3D). Research has demonstrated that the kidneys exhibit a high level of EGFR, and that diabetes may be prevented by blocking the EGFR signaling pathway [26].

The protein–protein interactions of overlapping genes (43) were displayed on the String database. EGFR, PI3KCA, and MAPK10 were shown to be the primary proteins of Sch A using molecular docking. In particular, the most crucial target for Sch A to alleviate DN was EGFR. Prior research has demonstrated the significance of EGFR in DN, and Sch A down-regulates EGFR to prevent cell migration and encourage death [36]. Similar to this, kidney healing following acute damage is aided by EGFR activation [37]. Further investigation revealed that renal fibrosis caused by unilateral ureteral obstruction [26], renal hypertension [38], subtotal nephrectomy [39], or angiotensin II/endothelin-triggered kidney damage [40] requires sustained EGFR activation as an essential step. Here, we demonstrated that elevated EGFR signaling causes renal fibrosis and apoptosis in diabetic rats. Notably, Sch A was able to effectively inhibit EGFR phosphorylation and shield the diabetic rats’ kidneys from injury.

The connection of PTRF, sometimes referred to as Cavin1, with caveolae could be regulated by its reversible phosphorylation [41]. Studies have demonstrated that upregulation of PTRF contributes to delayed wound healing associated with diabetes [42]. In addition, the EGFR/PI3K/AKT pathway controls the expression of PTRF [43]. Therefore, we proposed that alleviating diabetic nephropathy by Sch A might be somewhat dependent on the production of PTRF, which was achieved by blocking the EGFR tyrosine kinase. Here, we showed that PTRF and EGFR were related, and that DN had much higher levels of PTRF expression. Surprisingly, Sch A halted the pattern. Thus, we think that treating diabetic nephropathy may be affected by controlling PTRF expression by blocking the EGFR tyrosine kinase. However, whether Sch A protects against DM-induced kidney injury via modifying PTRF expression is uncertain and has to be further clarified.

A large number of studies indicated that intracellular mechanisms regulating cell proliferation, differentiation, and apoptosis, such as MAP kinases, JAK/STAT, src kinases, and PI3K/AKT pathways were activated by EGFR [44]. Since AKT is a direct target of EGFR, EGFR inhibition prevents AKT phosphorylation, which is necessary for the incidence and progression of DN [45]. Prior research has established that the down-regulation of PI3K/AKT activation leads to renal proximal tubular cell death and that the PI3K/AKT pathway is inhibited in DN animal kidneys [46,47]. By phosphorylating GSK-3β (Ser9), phosphorylated AKT functions as a negative regulator of GSK-3β [48]. AKT phosphorylation is suppressed in DN, activating GSK-3β [49]. The intrinsic apoptotic pathway is linked to active GSK-3β because it raises Bax and decreases Bcl-2 [27]. The treatment of DN model rats with Sch A dramatically increased the activity of AKT, blocked the activation of GSK-3β, and inhibited the apoptotic signaling pathway in diabetic renal tissues. Sch A modulated the Akt/GSK-3β pathway, which in turn prevents renal cell apoptosis.

In conclusion, Sch A has a protective effect on DN, EGFR may be a potential therapeutic target, and AKT/GSK-3β may be involved in this process. However, the limitations of these methods are inherent. First, the dosage of Schisandrin A should be further set to low, medium, and high doses, and the therapeutic effects of different medication times should be monitored. Second, we should knock down or add small molecule inhibitors in vitro experiments for further study.

## Figures and Tables

**Table 1:** The binding energy of Sch A and the hub proteins.

Protein	PDB ID	Affinity kcal·mol^−1^	Amino Acid Residue	Interaction
EGFR	3w2s	−7.18	ASP-A:800	Conventional hydrogen bond
PIK3CA	7l1c	−6.73	ILE-B:7; PRO-B:5	Conventional hydrogen bond
MAPK10	2b1p	−6.58	GLN-A:155; ASN-A:152	Conventional hydrogen bond

## Data Availability

All datasets generated for this study are included in the manuscript.

## Supplementary Materials

The following supporting information can be downloaded at: https://www.mdpi.com/article/10.3390/biology13080597/s1, Western Blot for Figure 5, Figure 6 and Figure 7.
