# Supplementary material for: Schisandrin A Attenuates Diabetic Nephropathy via EGFR/AKT/GSK3β Signaling Pathway Based on Network Pharmacology and Experimental Validation
Source: Biology (Basel). 2024 Aug 8;13(8):597. doi: 10.3390/biology13080597 (PMC11351691; doi:10.3390/biology13080597)

Figure 5C EGFR EXP001

20230828

20230829

p-EGFR

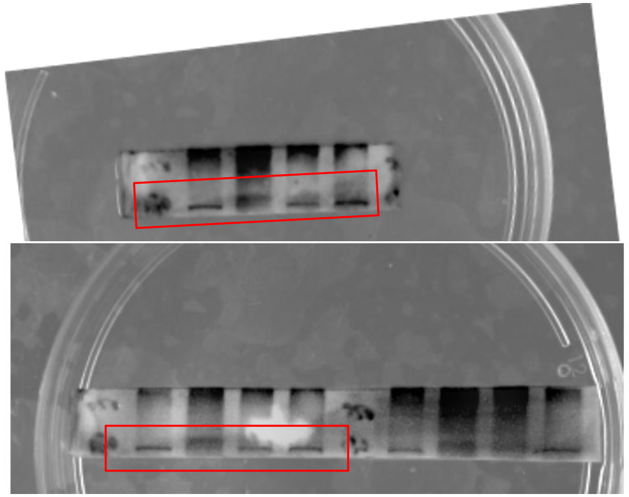

GAPDH

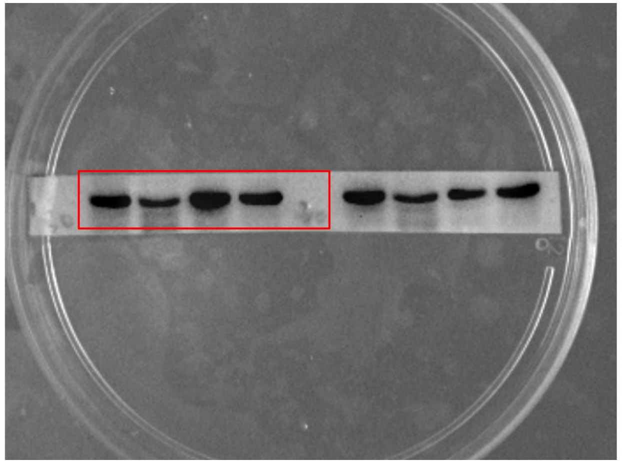

EGFR

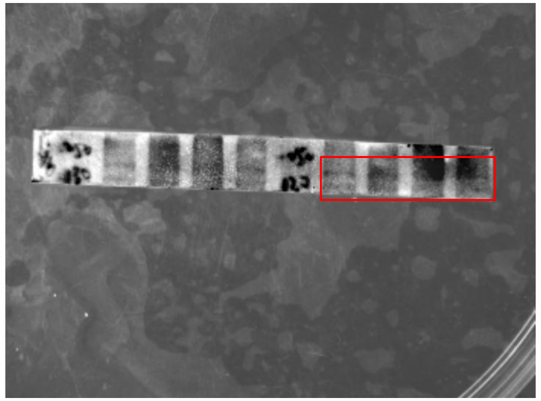

GAPDH

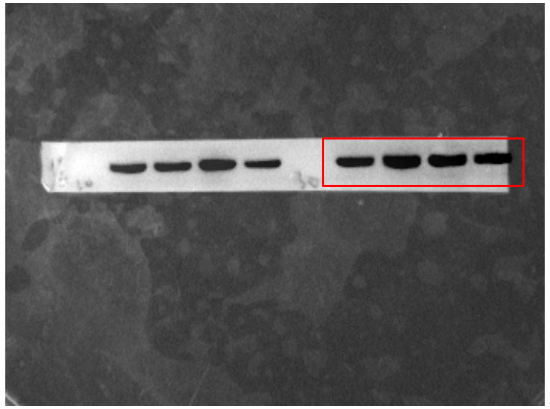

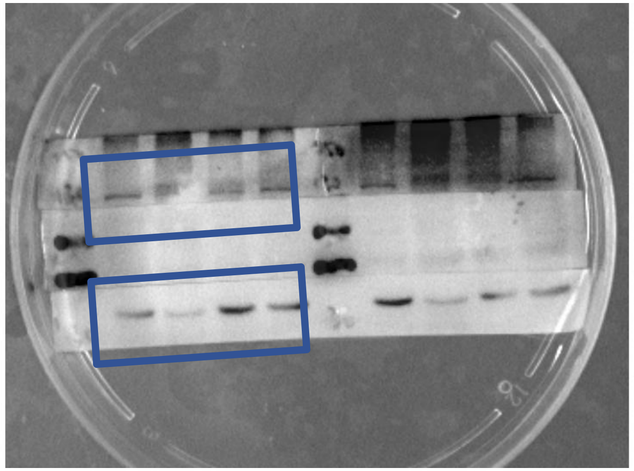

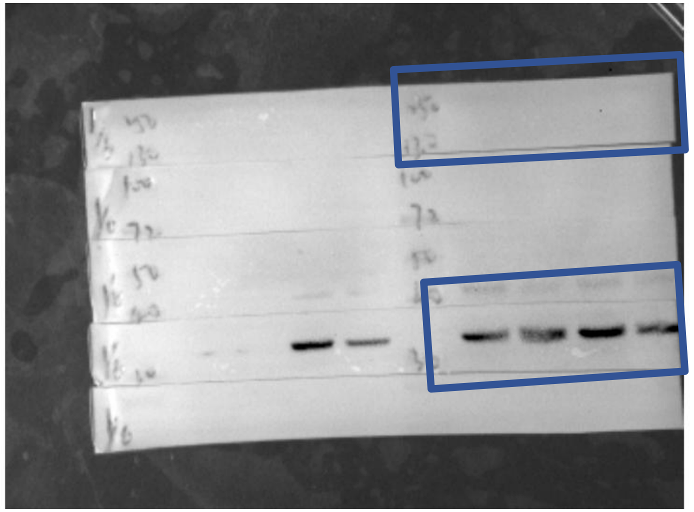

Figure 5C EGFR EXP002-003

20230905 EXP002-003

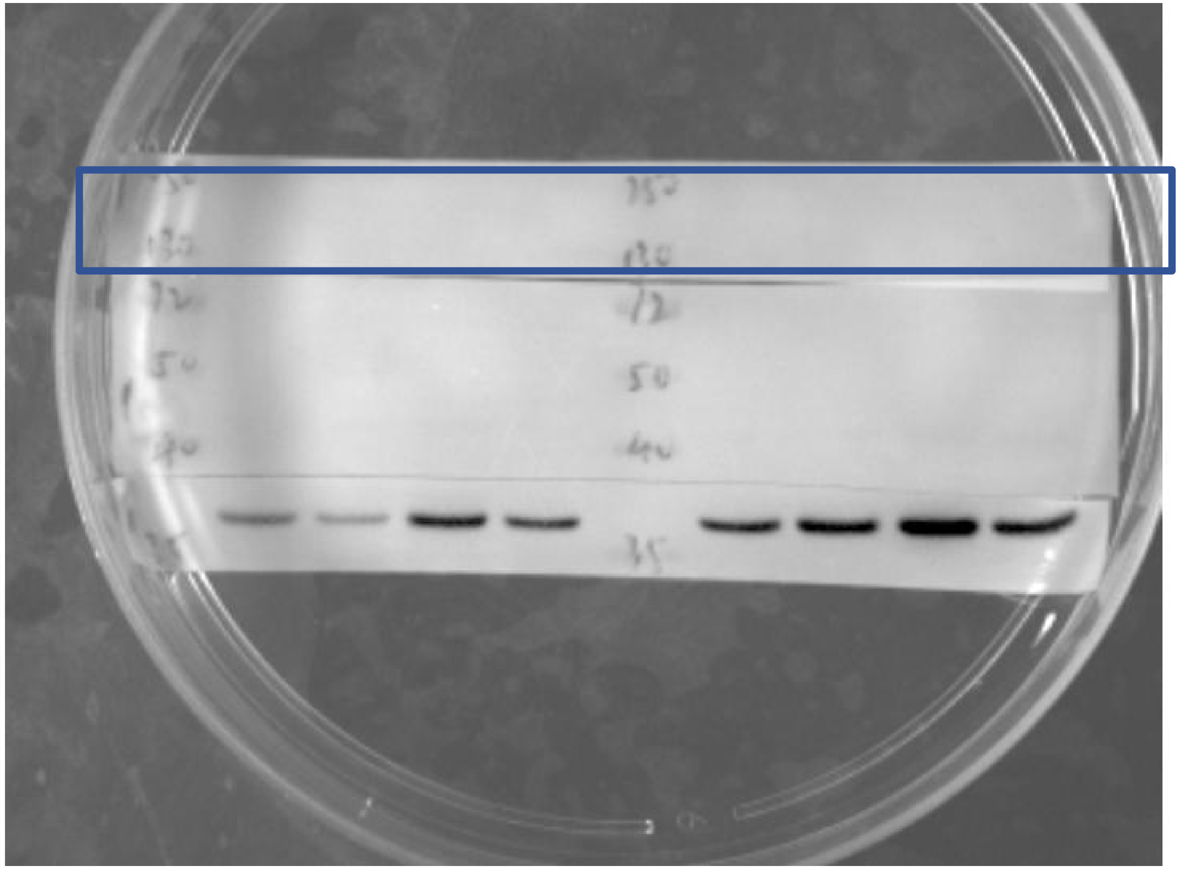

p-EGFR

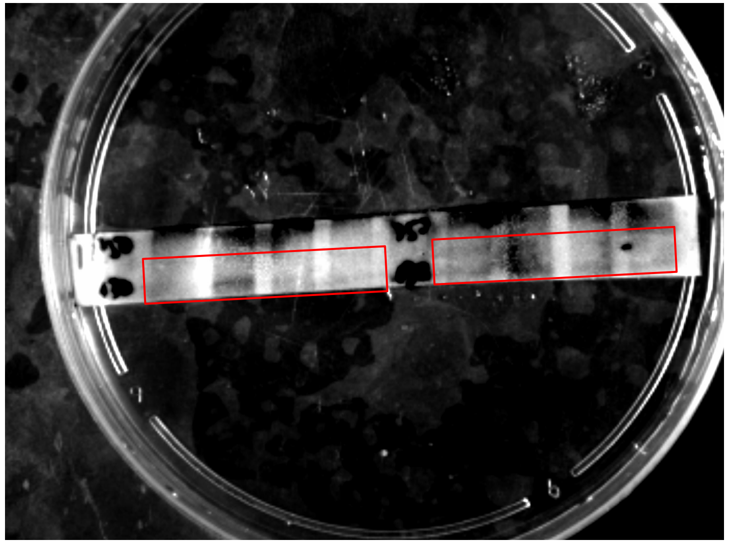

EGFR

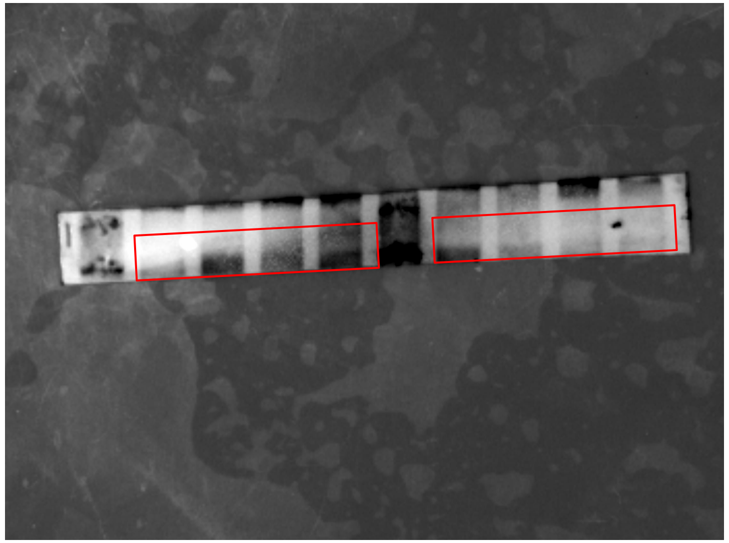

Figure 6C p-AKT EXP001-2

0829 membrane 1

P-AKT

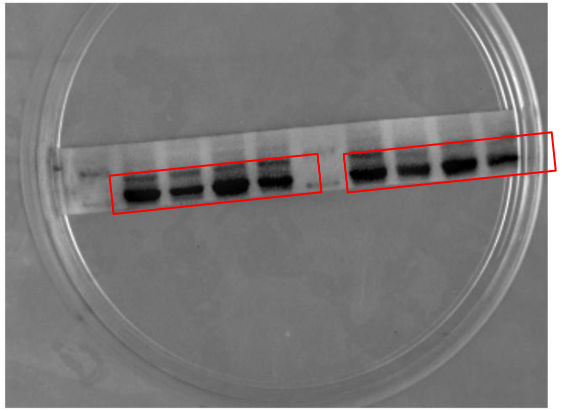

GAPDH

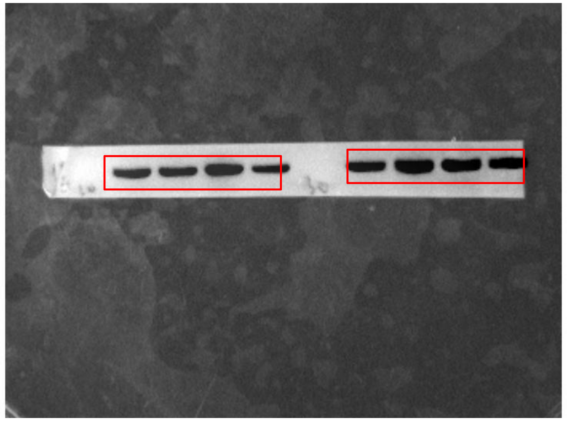

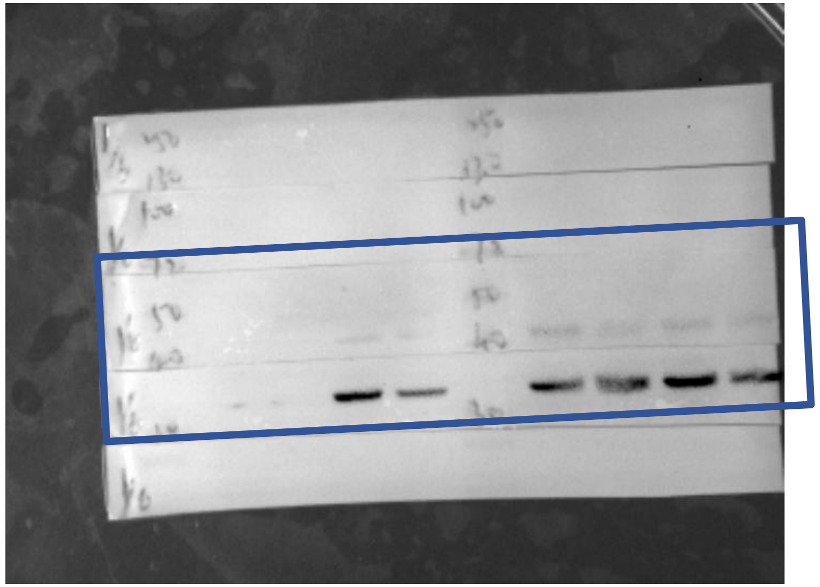

20230828 membrane 1

AKT

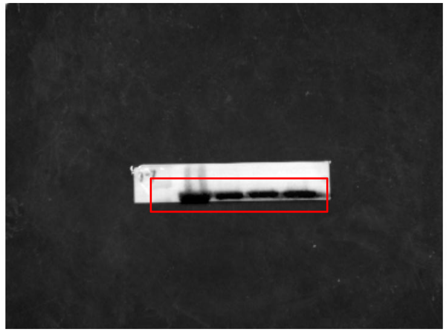

AKT

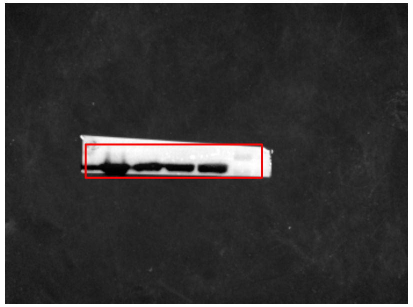

GAPDH

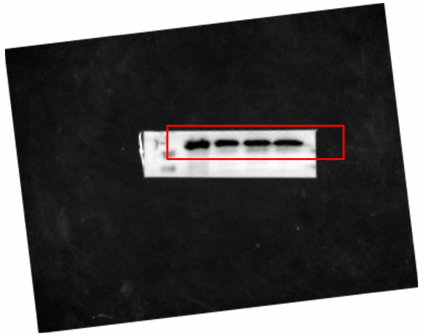

GAPDH

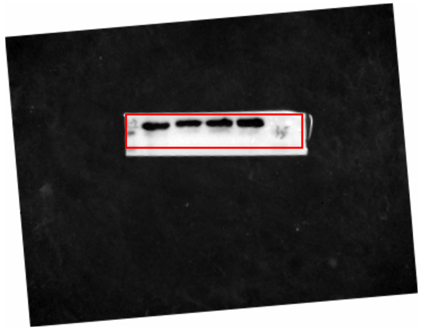

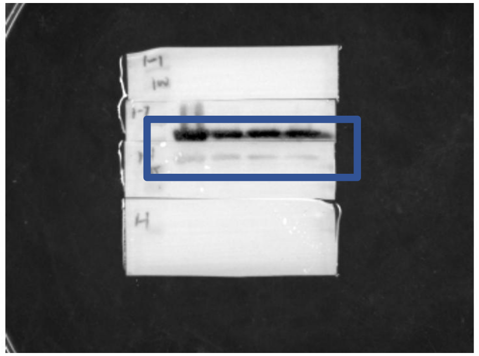

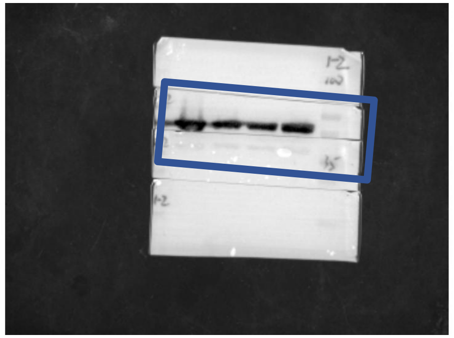

Figure 6C p-AKT EXP003-4

0830

P-AKT

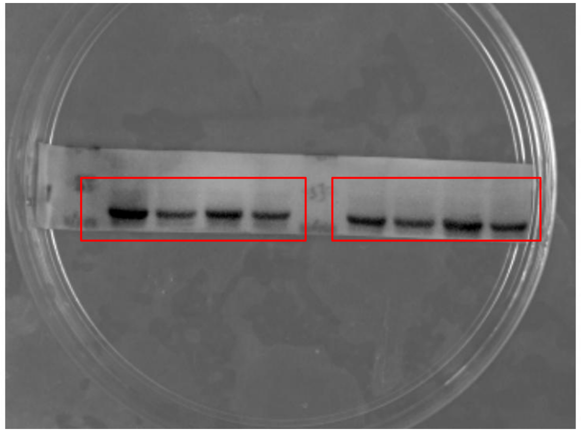

GAPDH

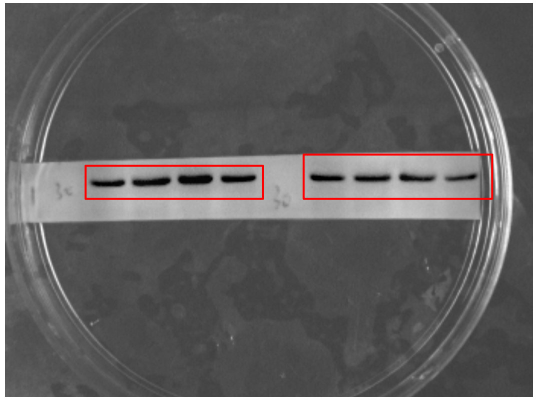

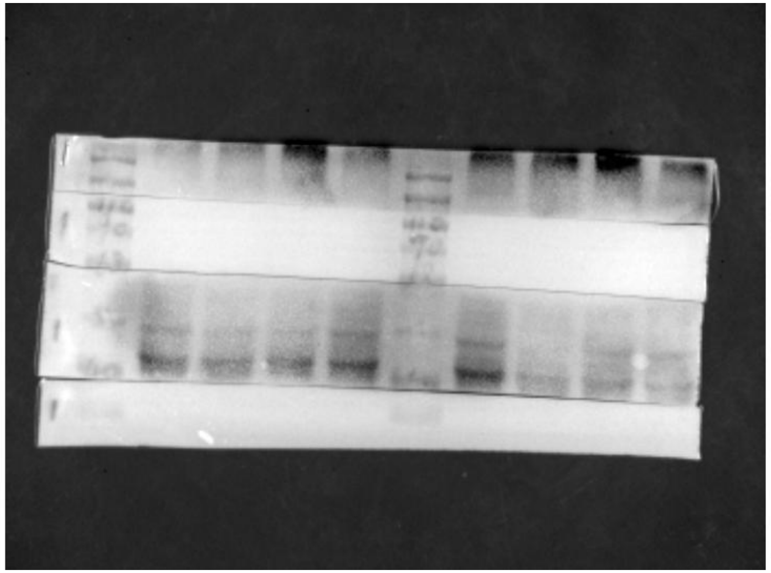

20230827 membrane 2

AKT

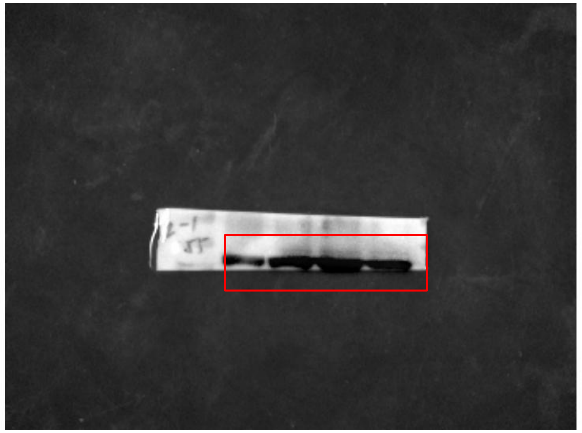

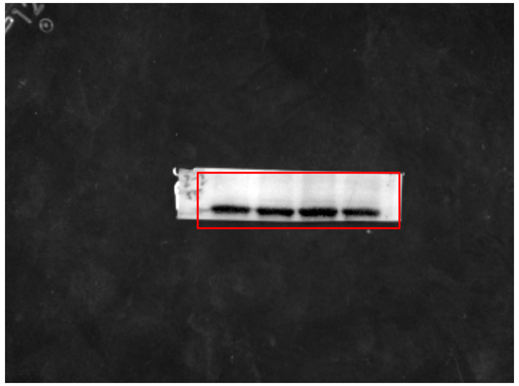

GAPDH

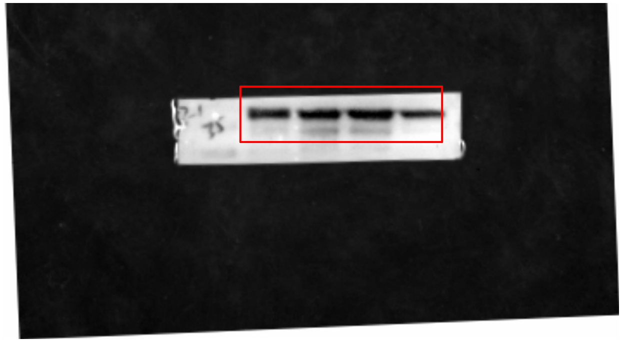

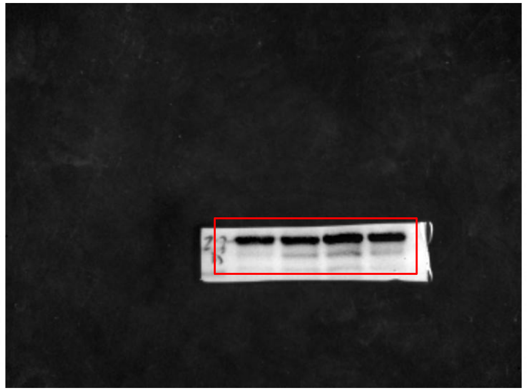

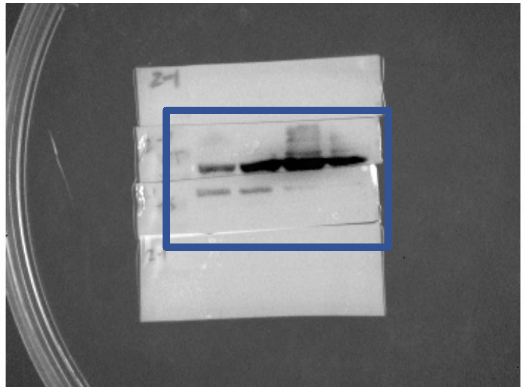

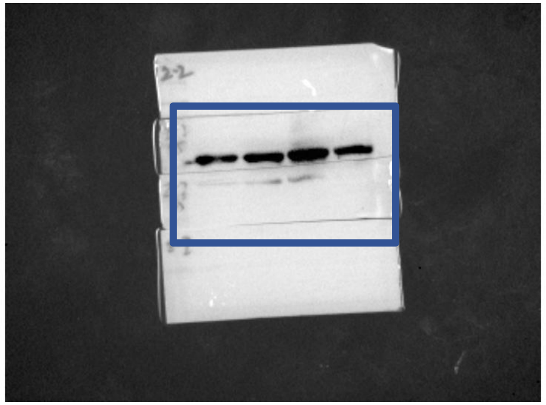

Figure 6C p-GSK3β EXP001

0826

p-GSK3B

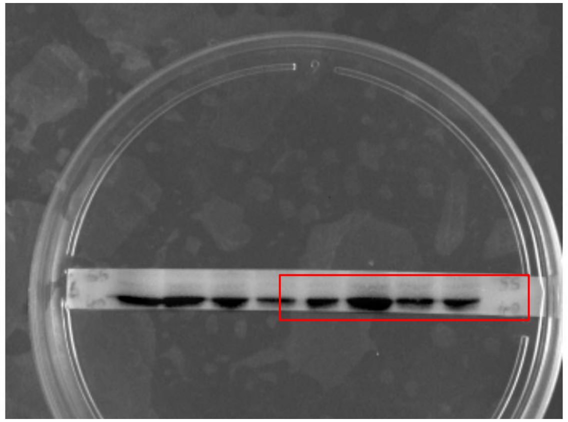

GAPDH

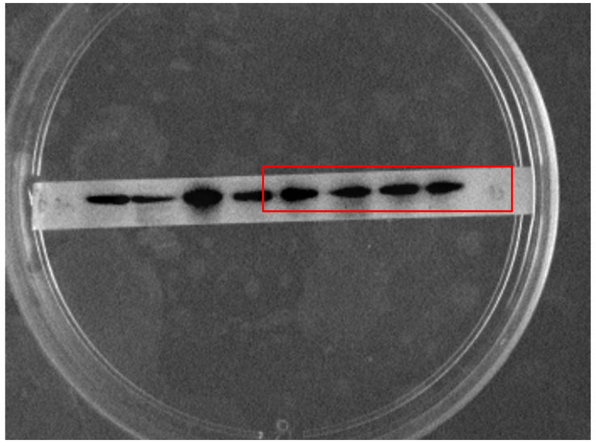

0717

GSK3B

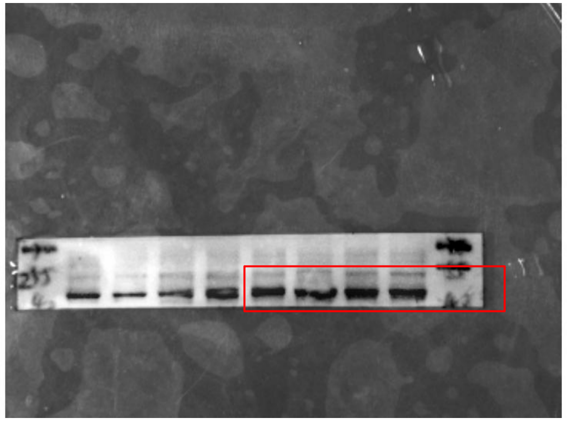

GAPDH

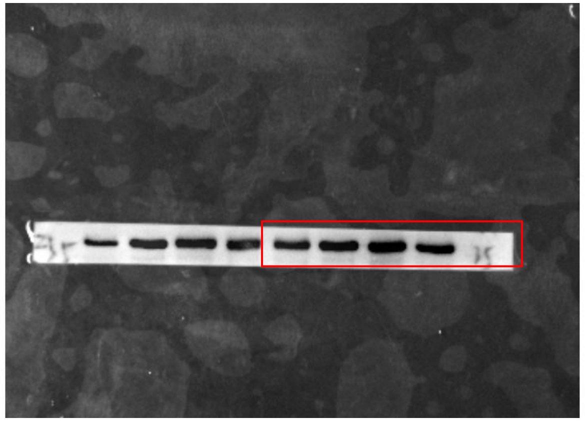

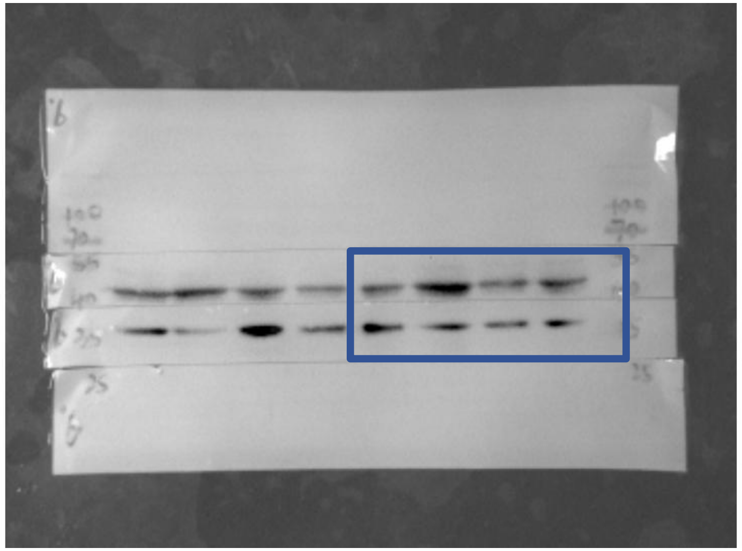

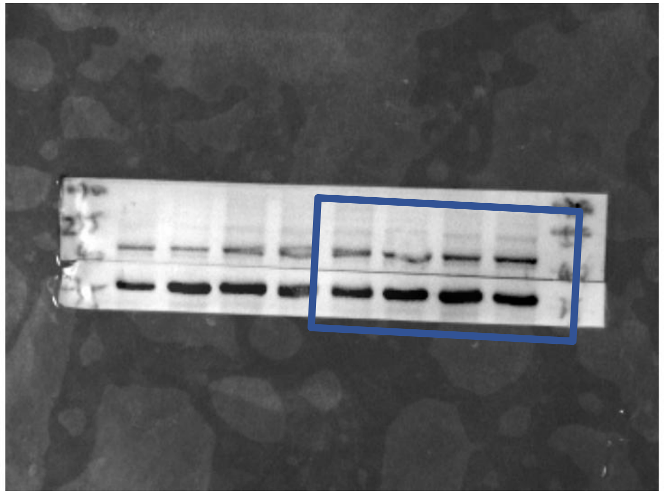

Figure 6C p-GSK3β EXP002-3

0824

p-GSK3B

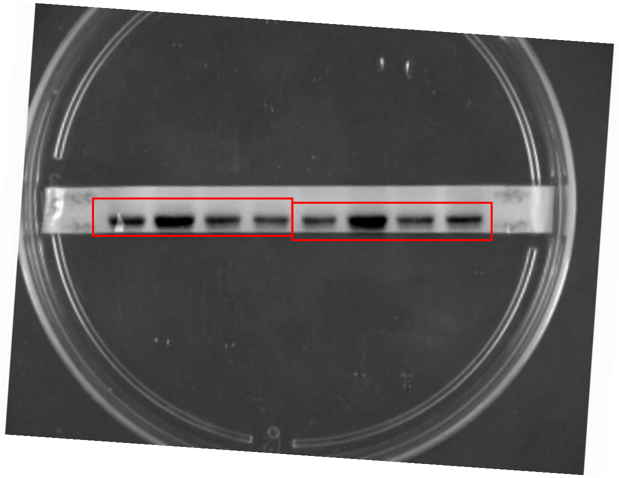

GAPDH

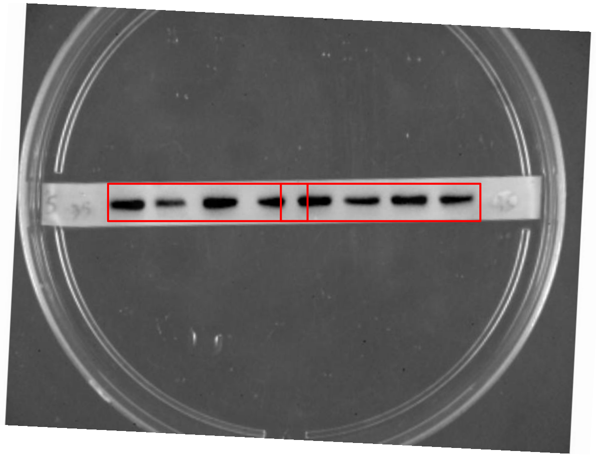

GSK3B

0718 membrane 2

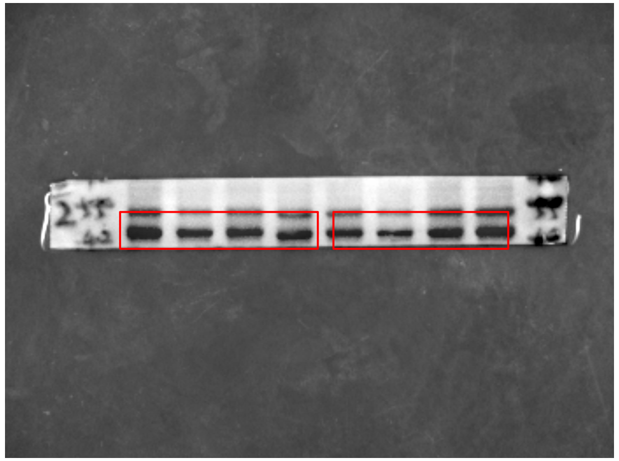

GAPDH

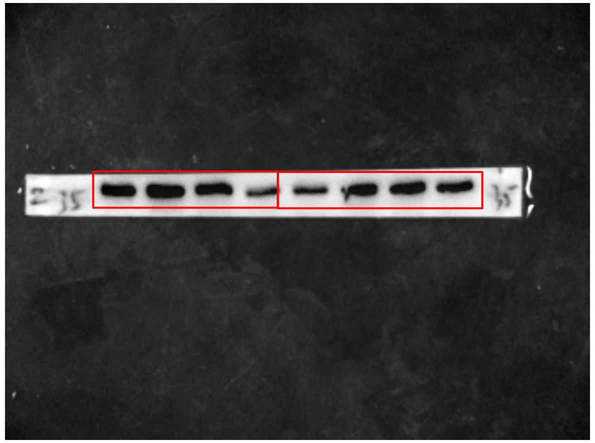

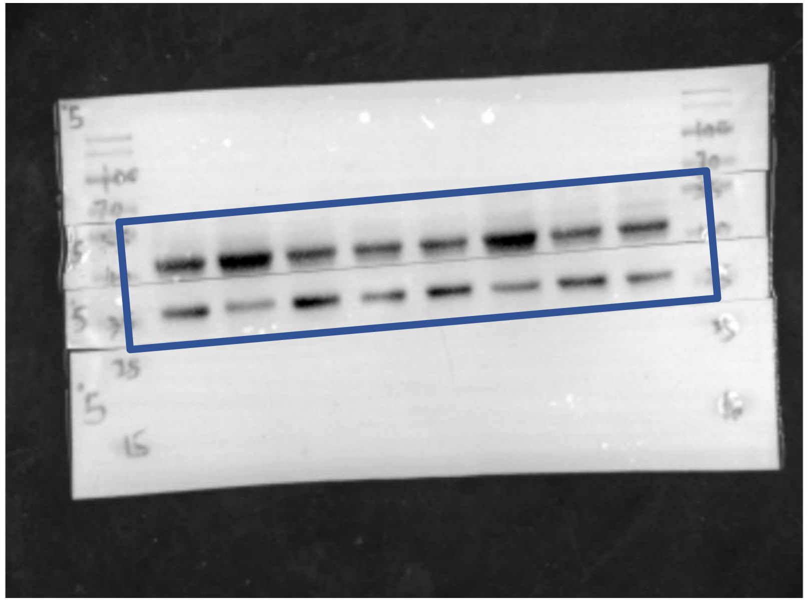

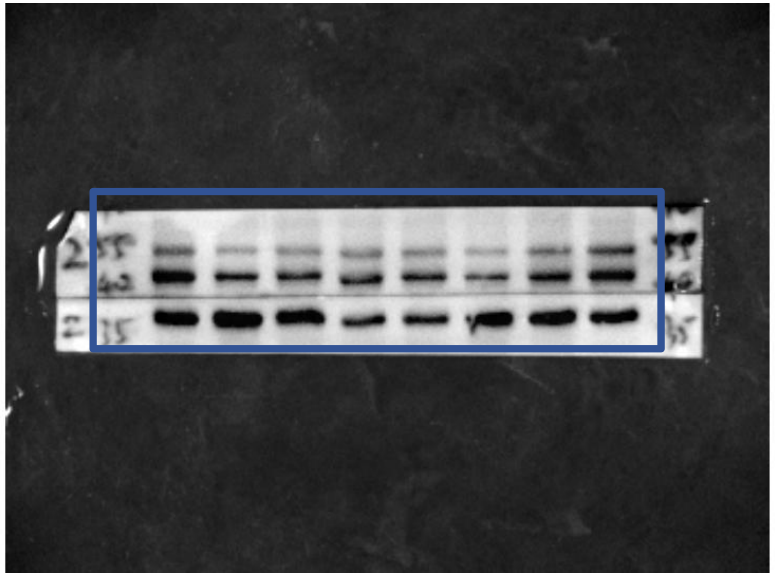

Figure 6D BCL2/BAX EXP001

20240122 membrane 2

20240121 membrane 1

BAX

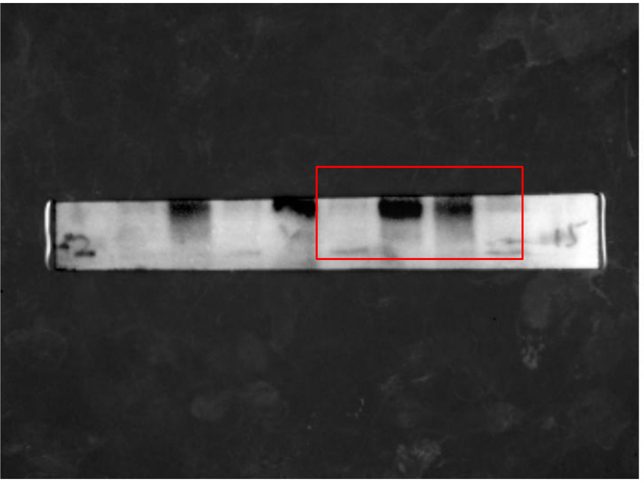

β-actin

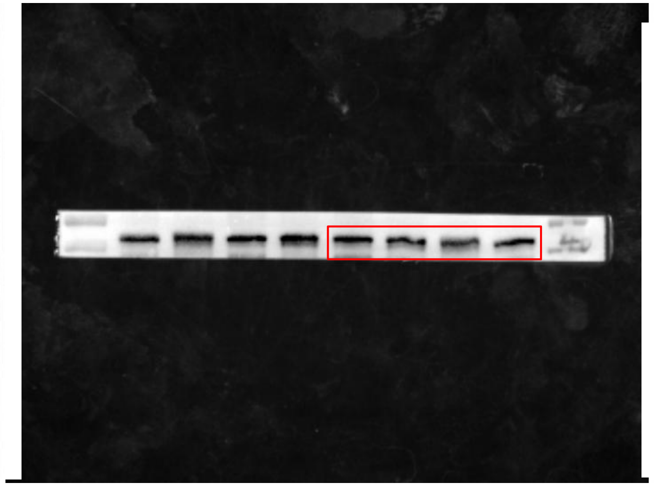

Bcl2

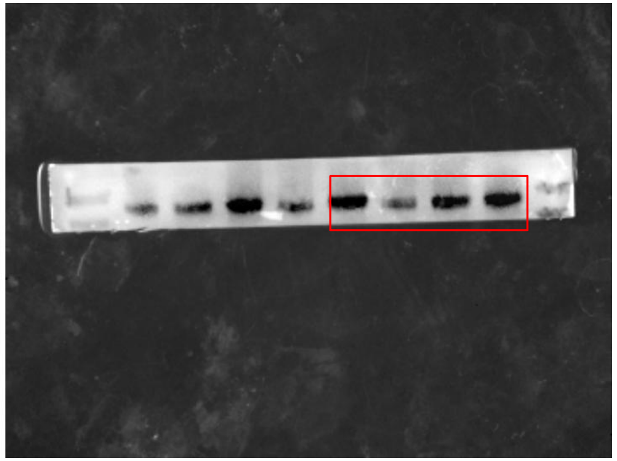

β-actin

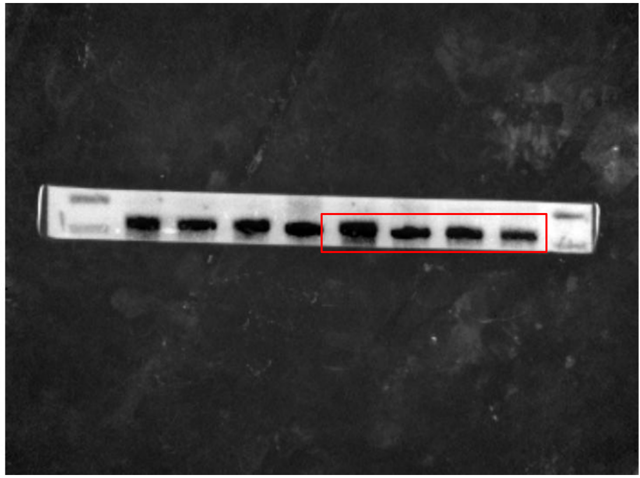

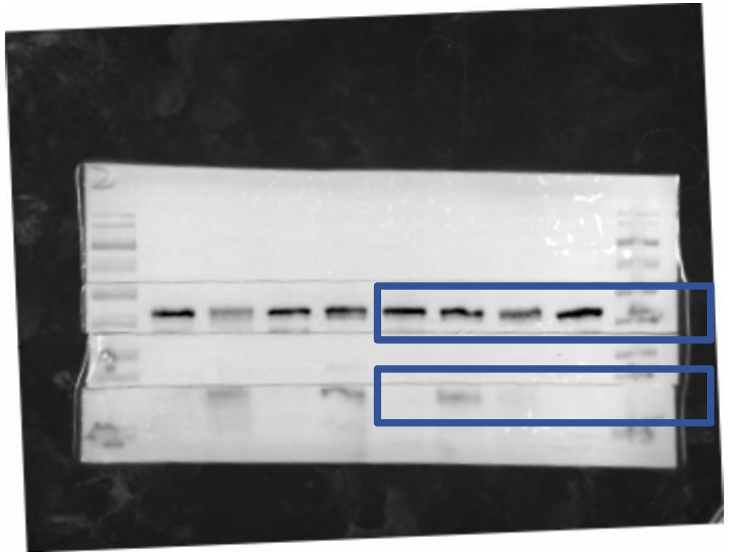

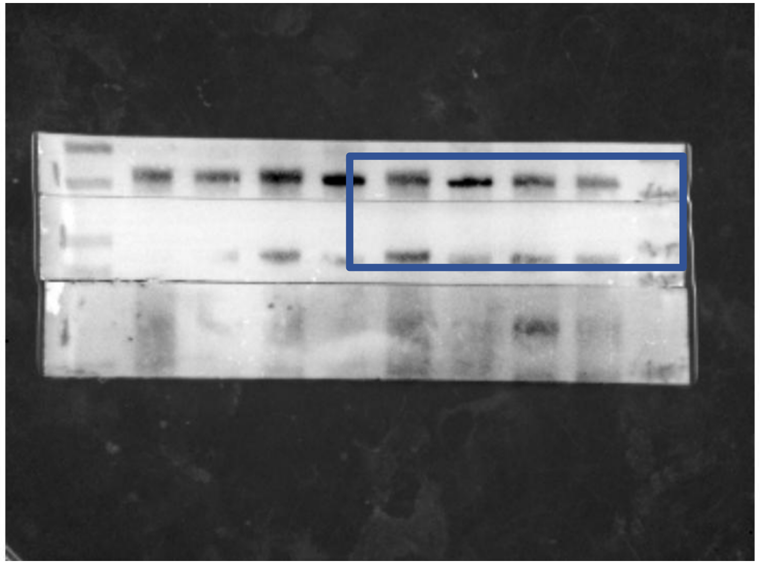

Figure 6D BCL2/BAX EXP002

20240123 membrane 4

BAX

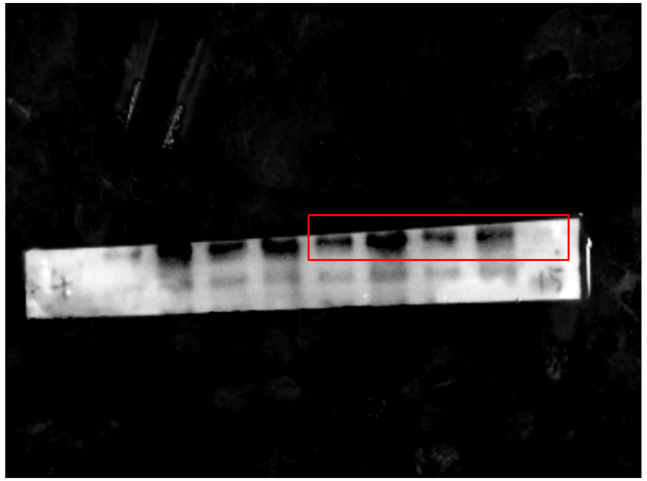

β-actin

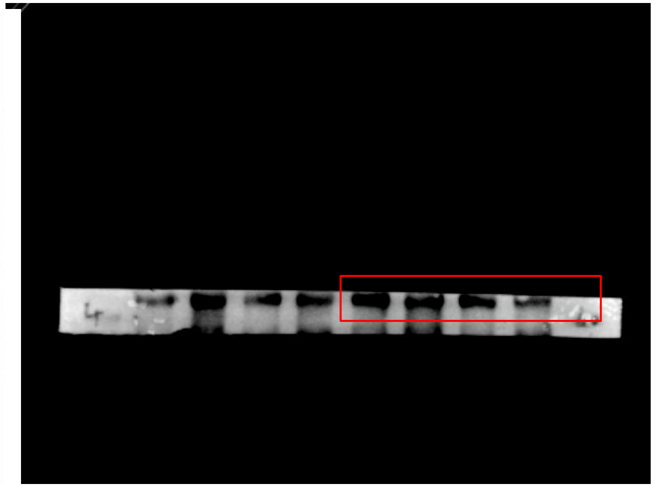

20240123 membrane 2

Bcl2

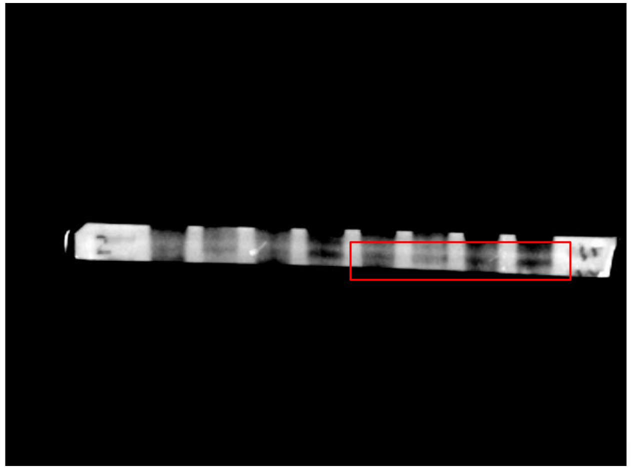

β-actin

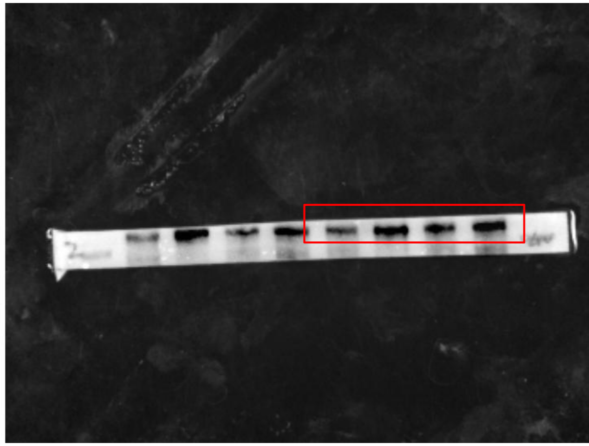

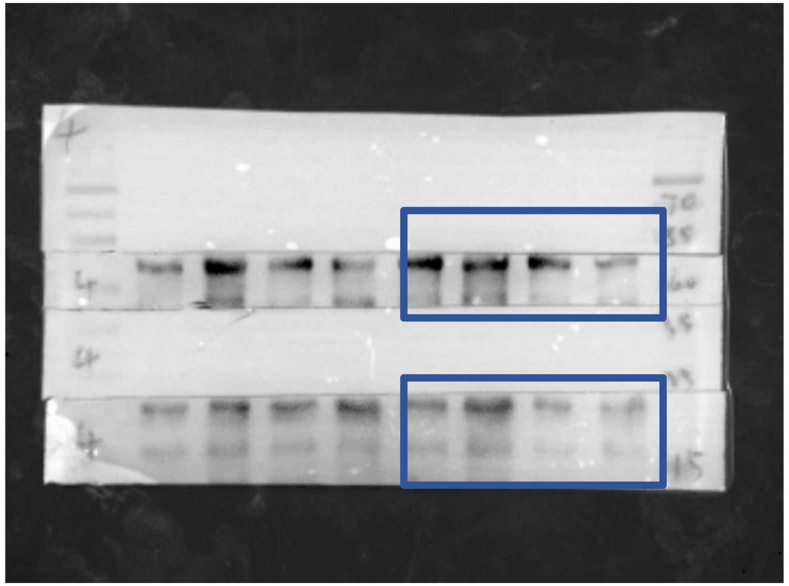

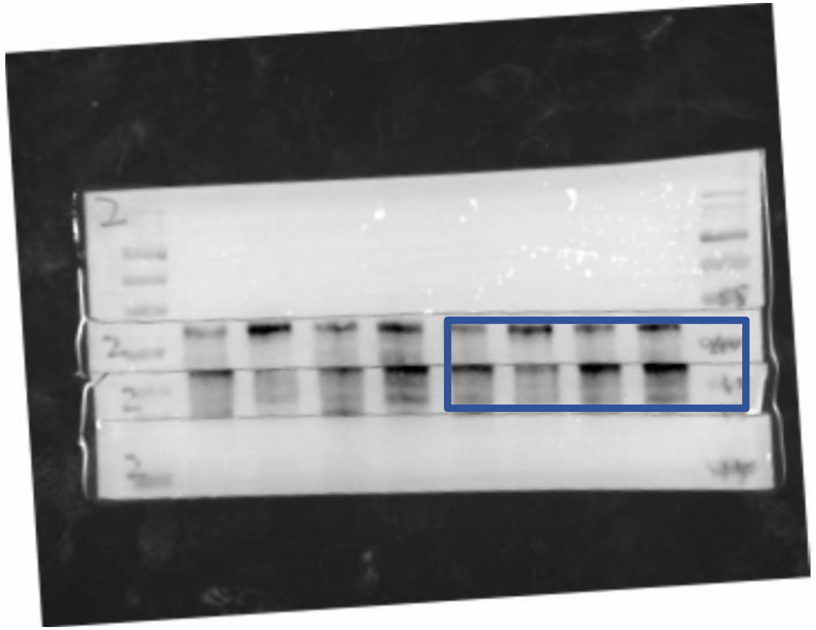

Figure 6D BCL2/BAX EXP003

20240122 membrane 1

Bcl2

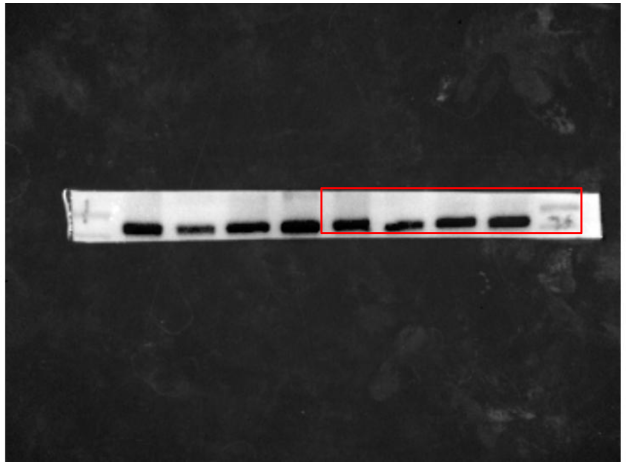

β-actin

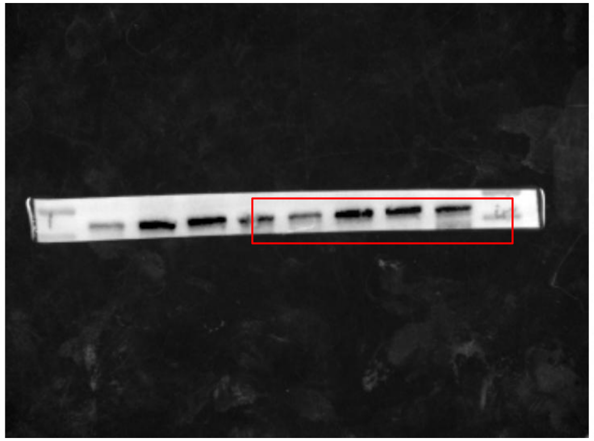

20240125 membrane 1

BAX

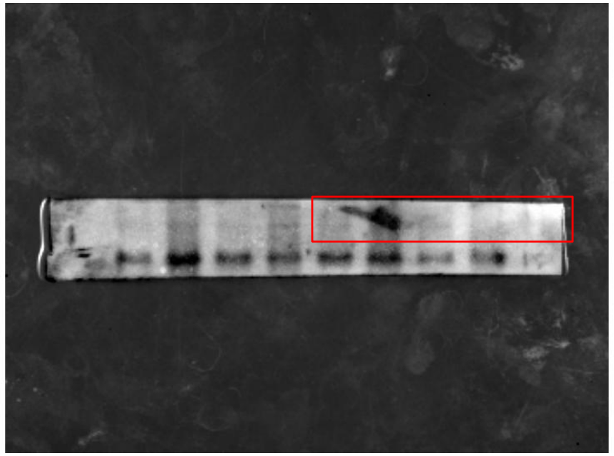

β-actin

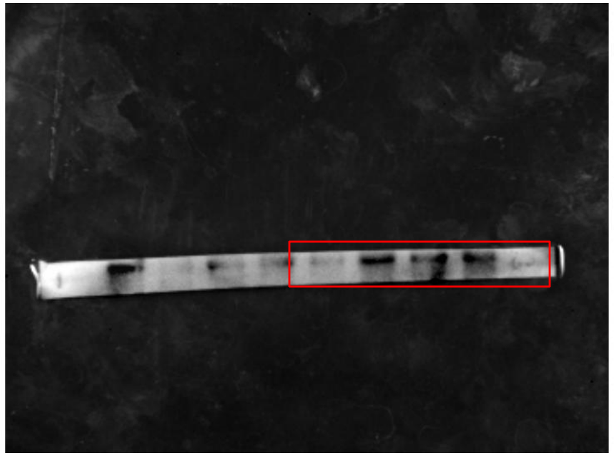

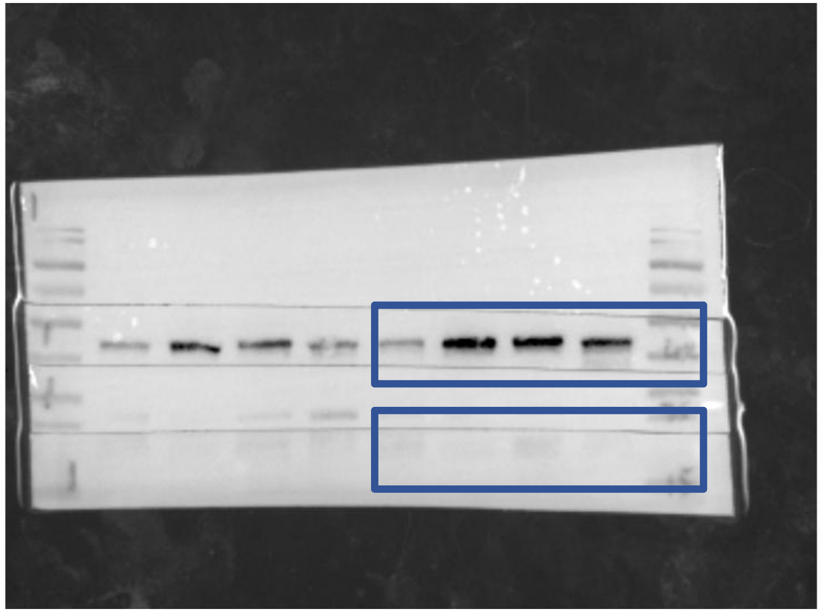

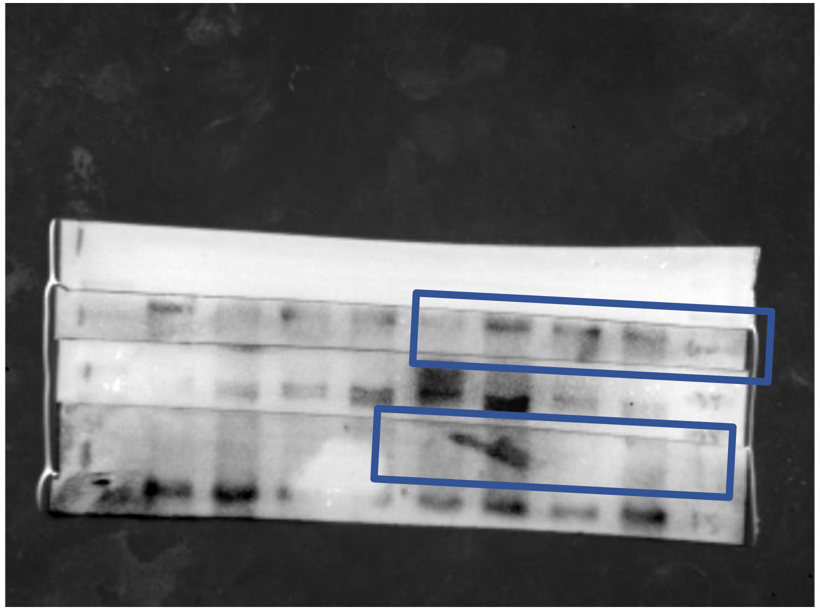

Figure 6D Cleaved-caspase3/caspase3 EXP001-2

20240116

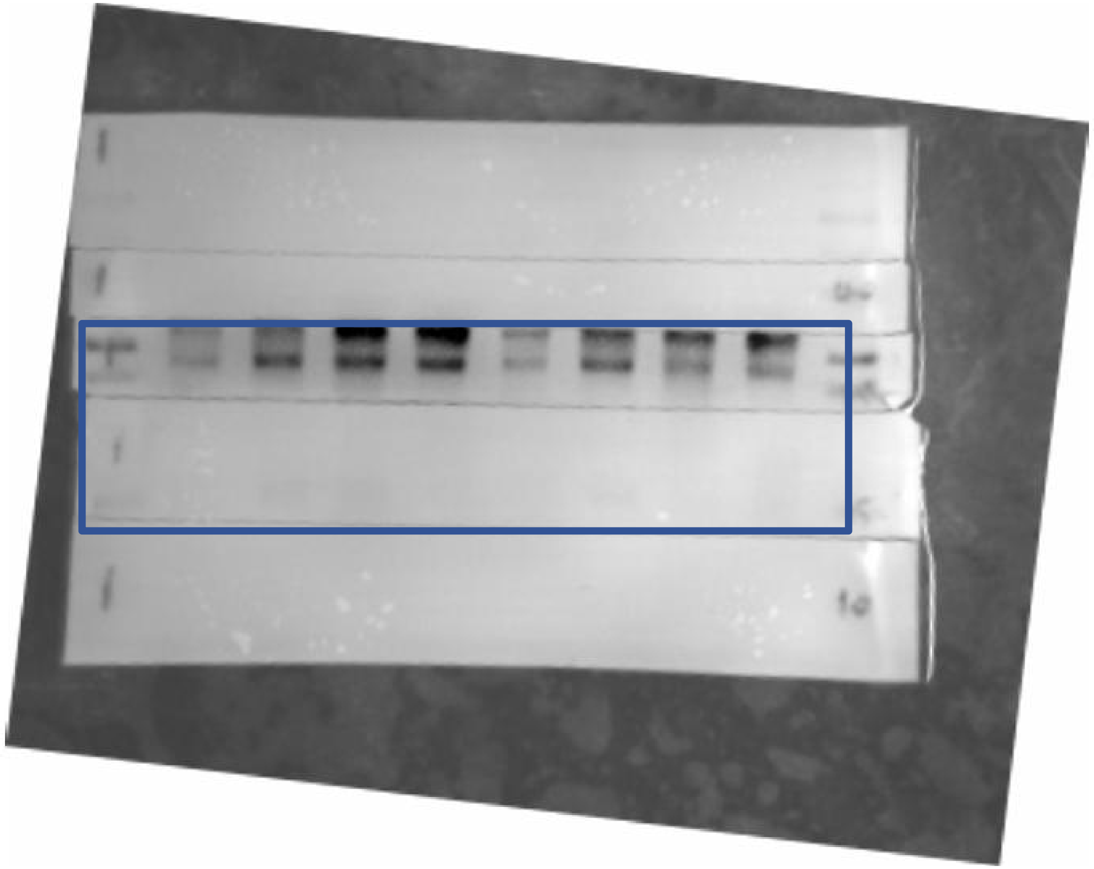

Cleaved-caspase3

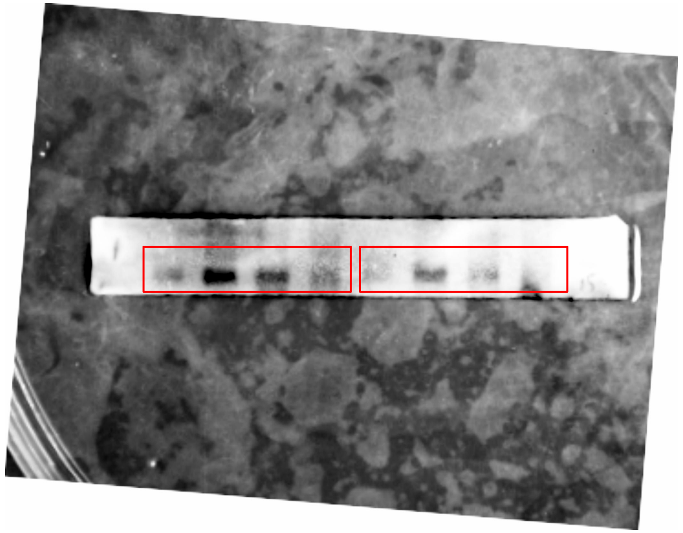

caspase3

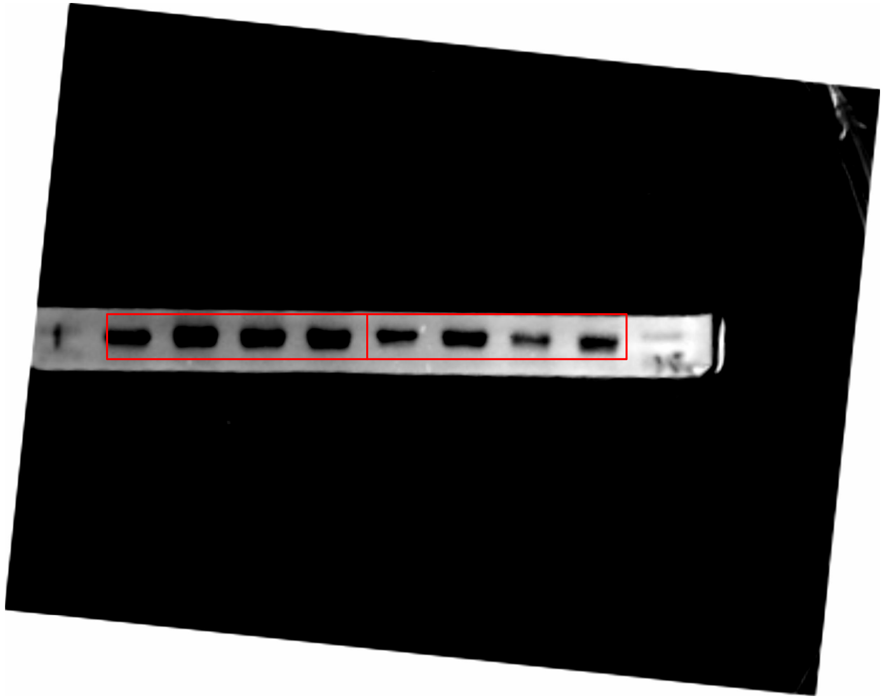

Figure 6D Cleaved-caspase3/caspase3 EXP003

20240118

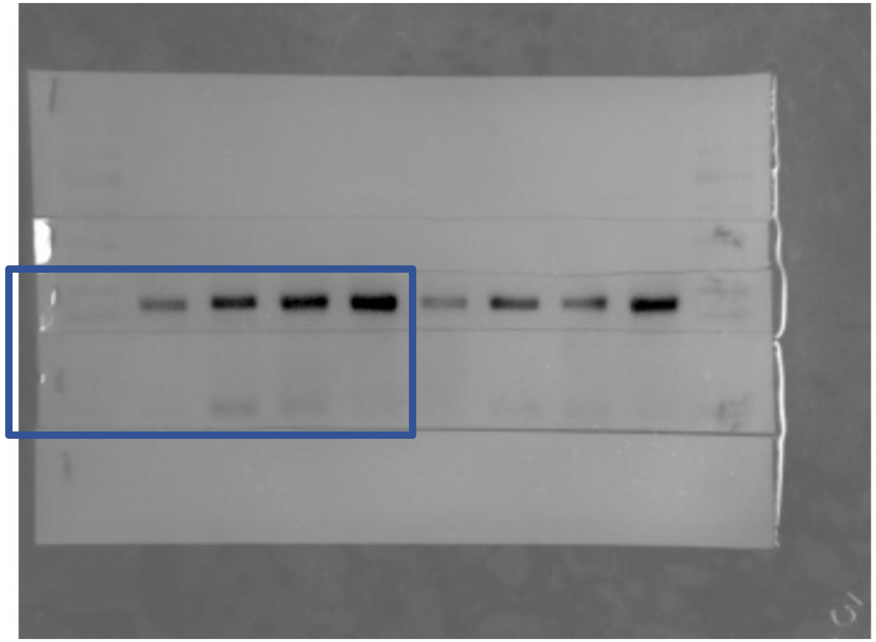

Cleaved-caspase3

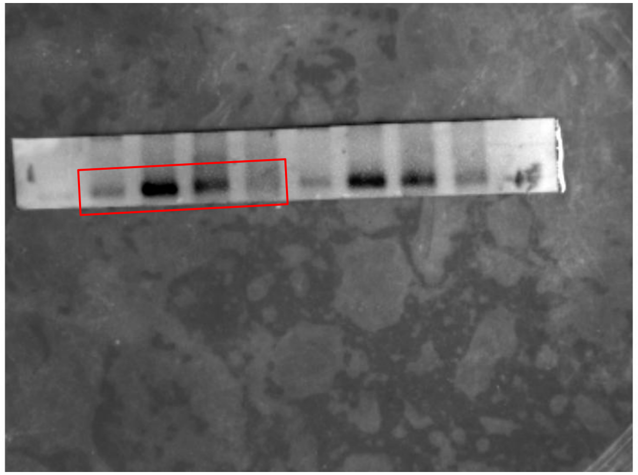

caspase 3

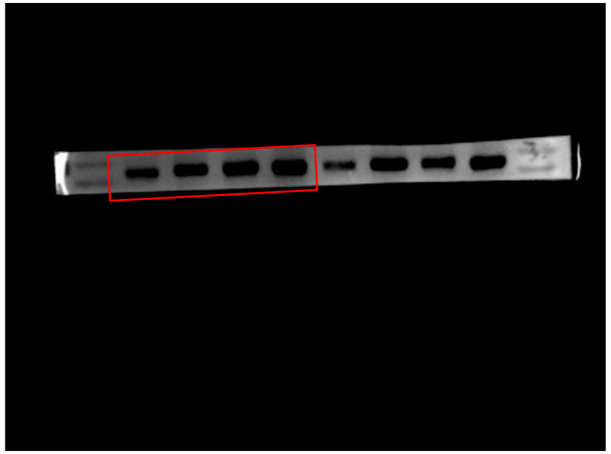

Figure 6D Cleaved-caspase3/caspase3 EXP004

20240120

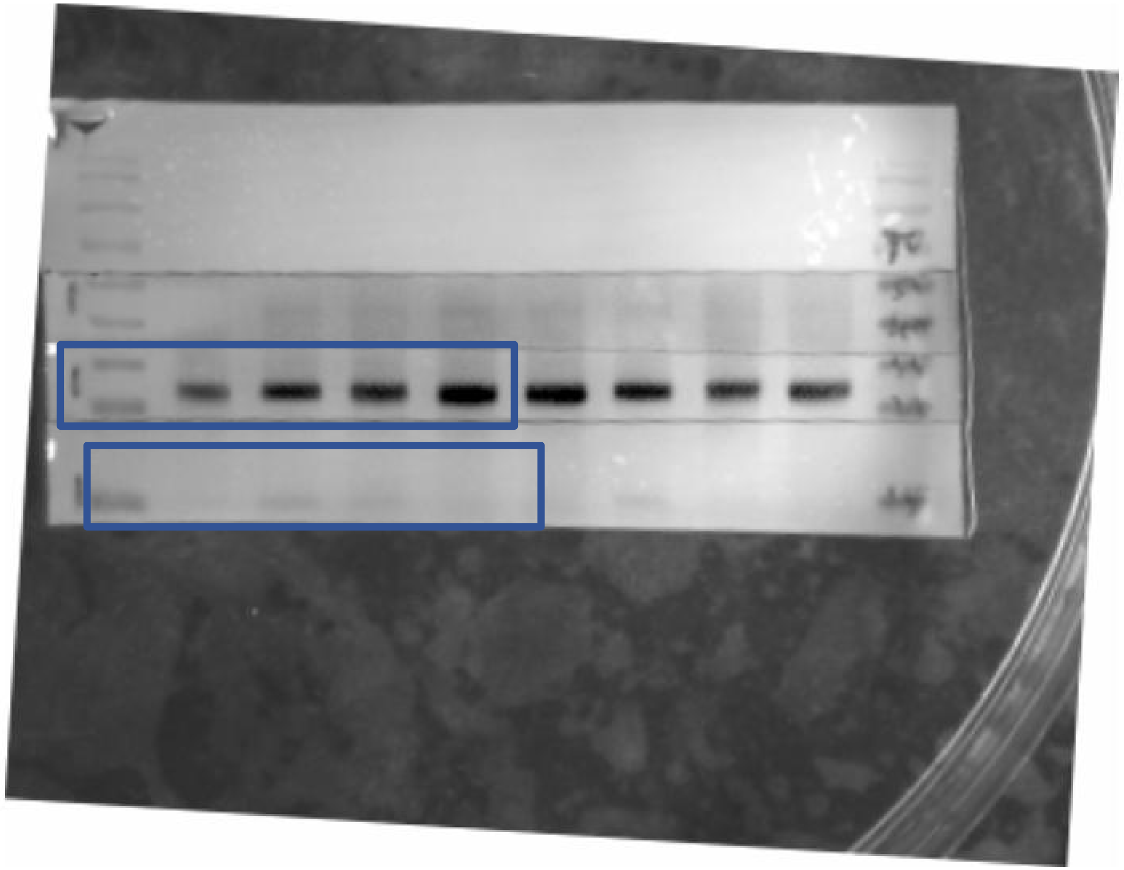

Cleaved-caspase3

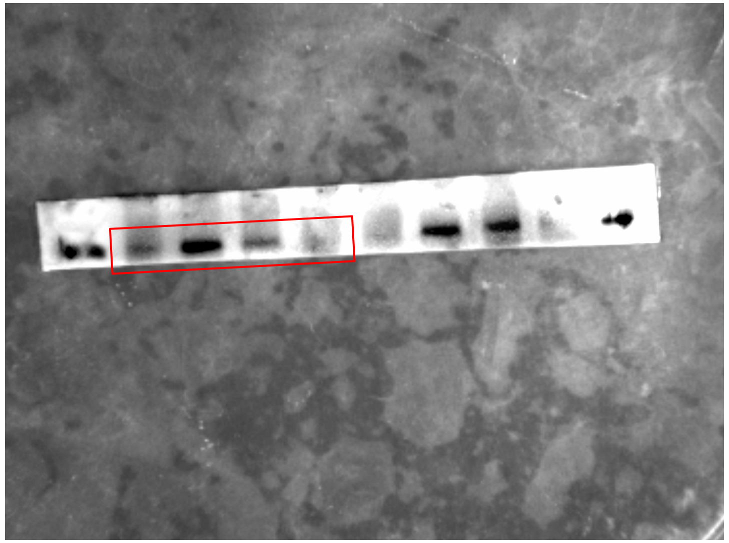

caspase 3

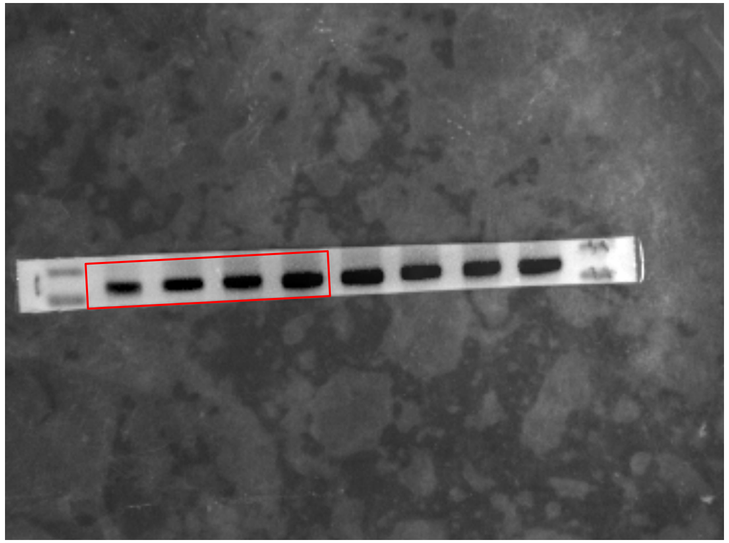

Figure 6D Cleaved-caspase3/caspase3 EXP005

20240125

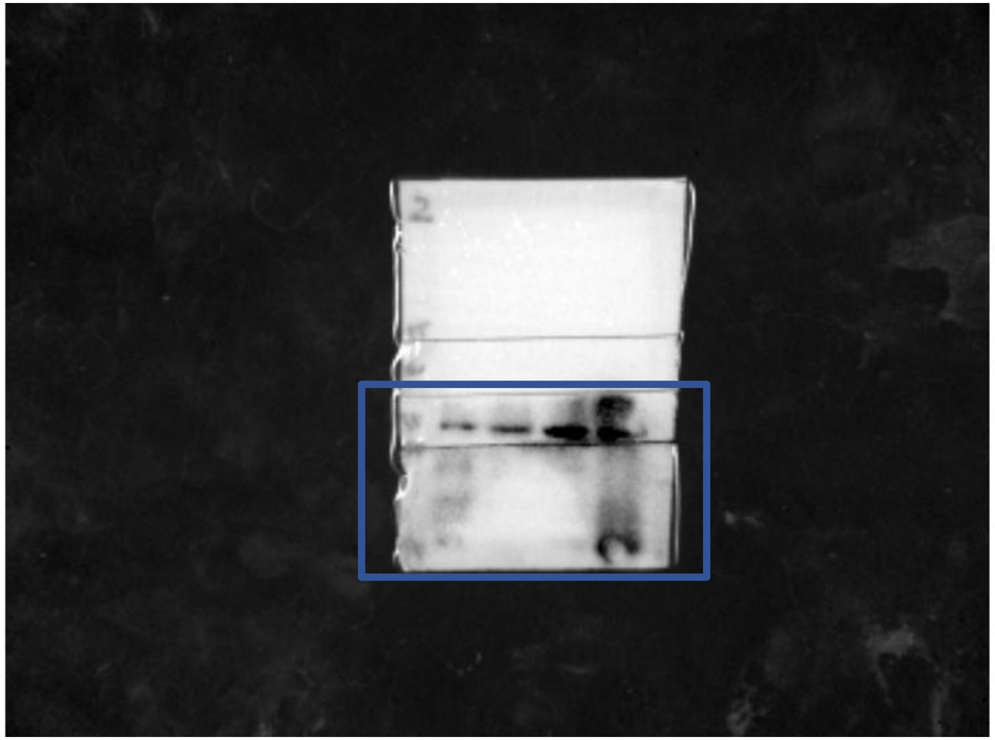

Cleaved-caspase3

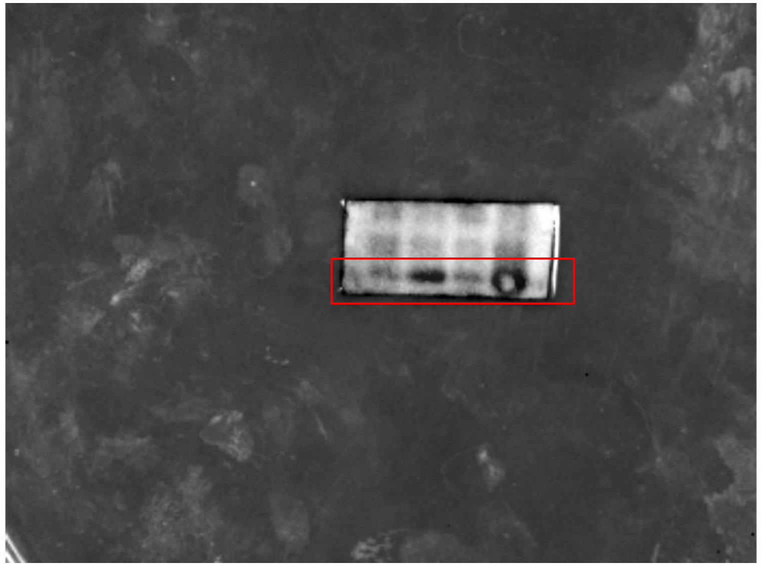

caspase 3

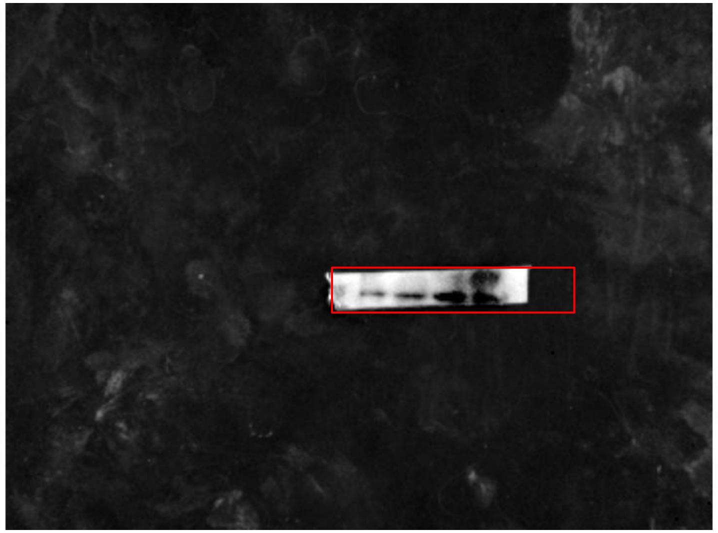

Figure 7D PTRF EXP001-2

20230817 membrane 5

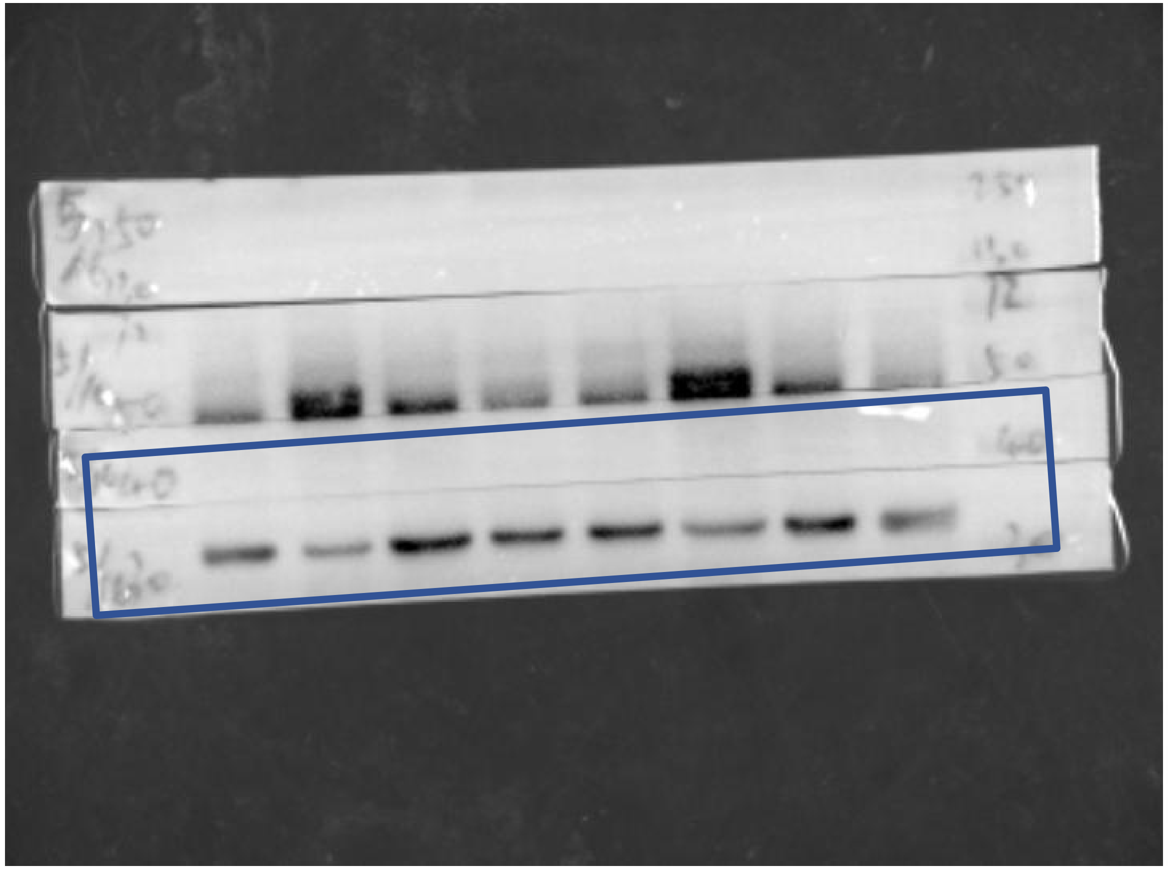

PTRF

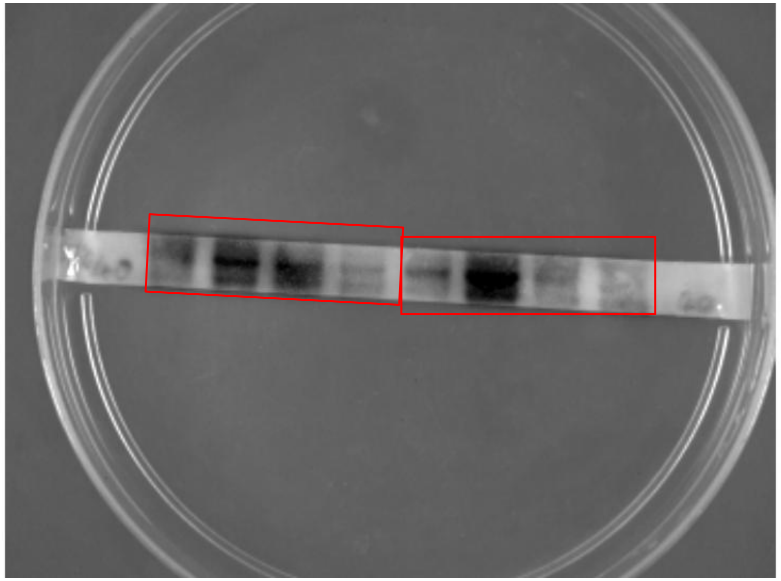

GAPDH

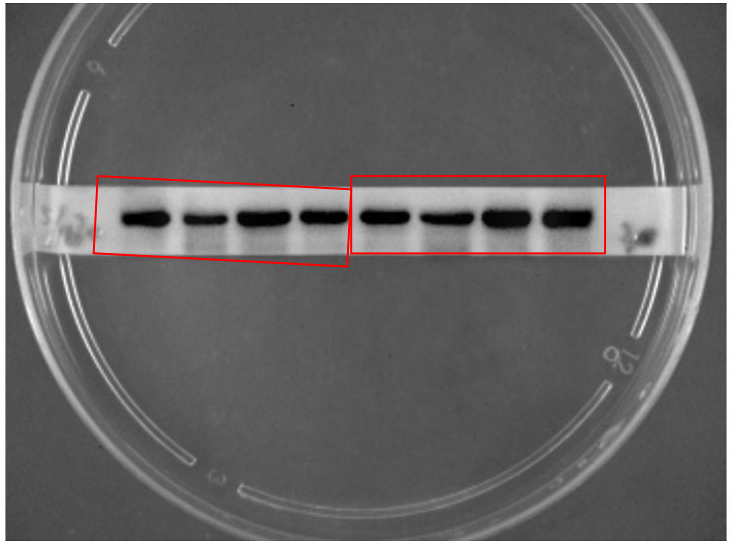

Figure 7D PTRF EXP003-4

20230817 membrane 6

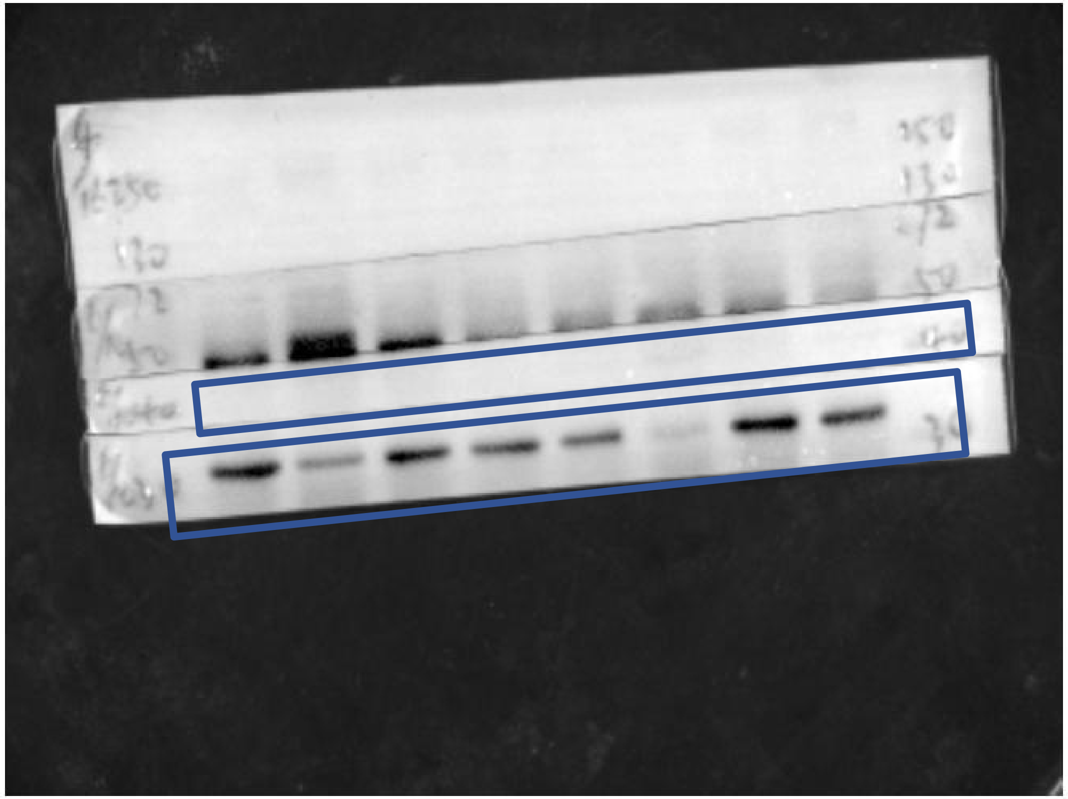

PTRF

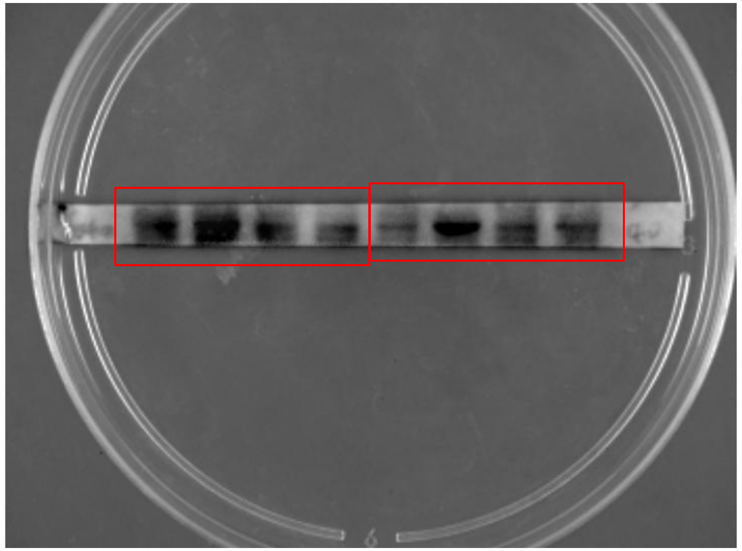

GAPDH

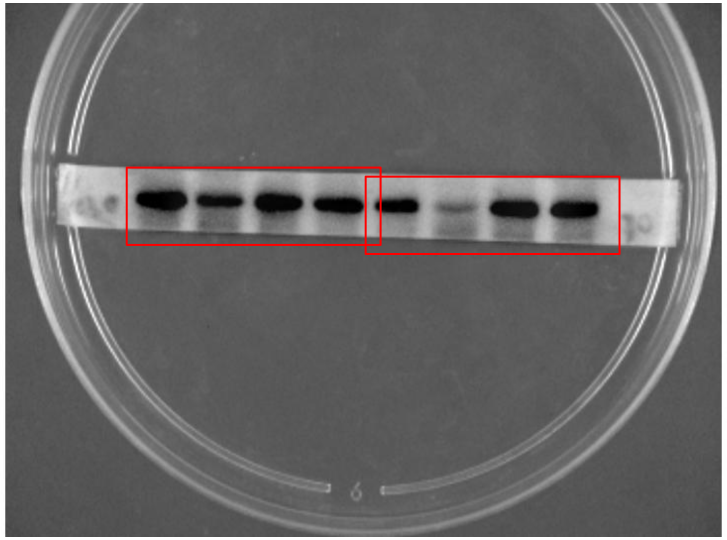

Figure 7D PTRF EXP005

20230823 membrane 6

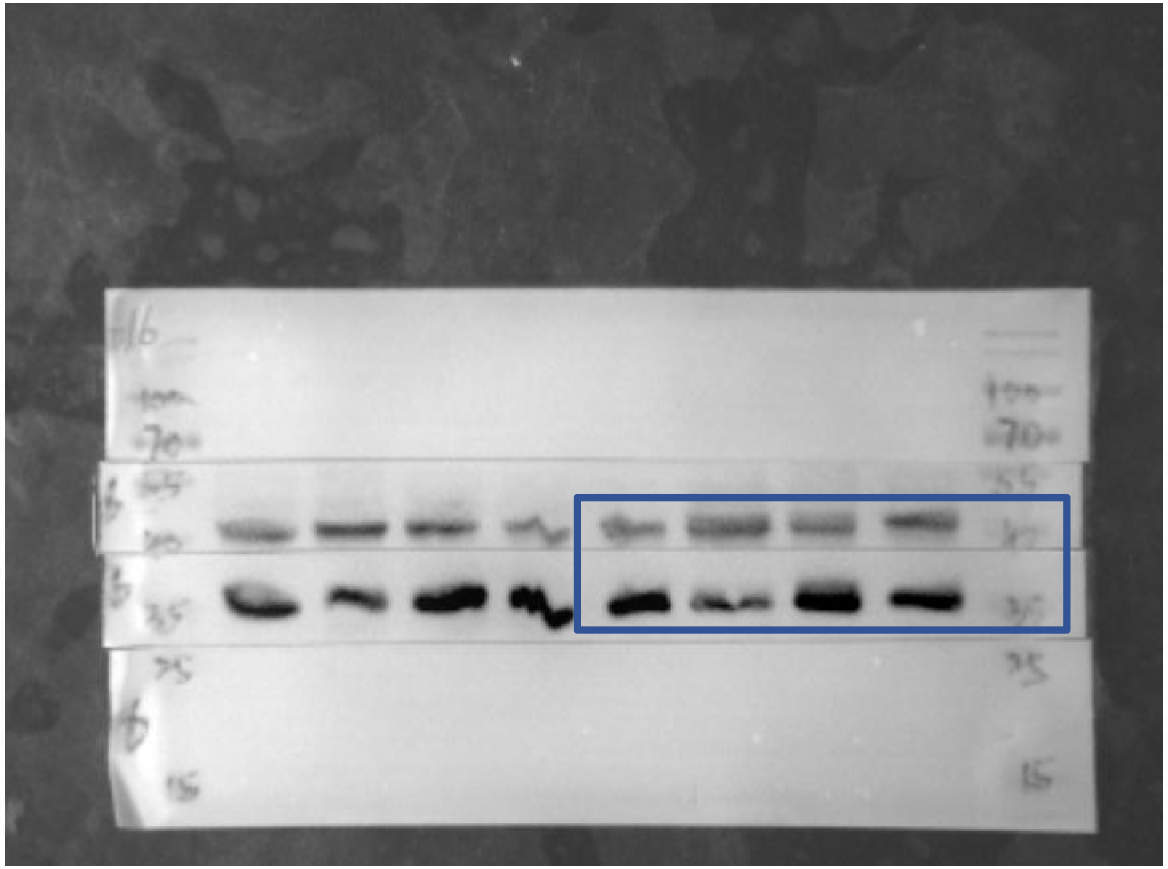

PTRF

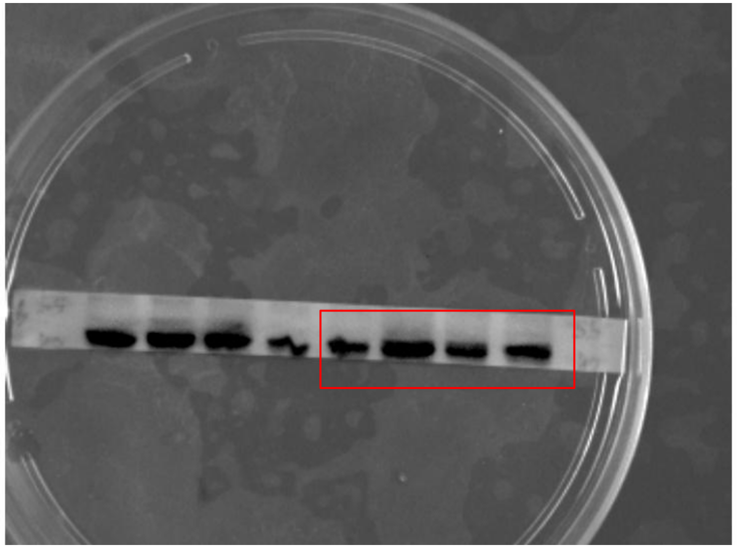

GAPDH

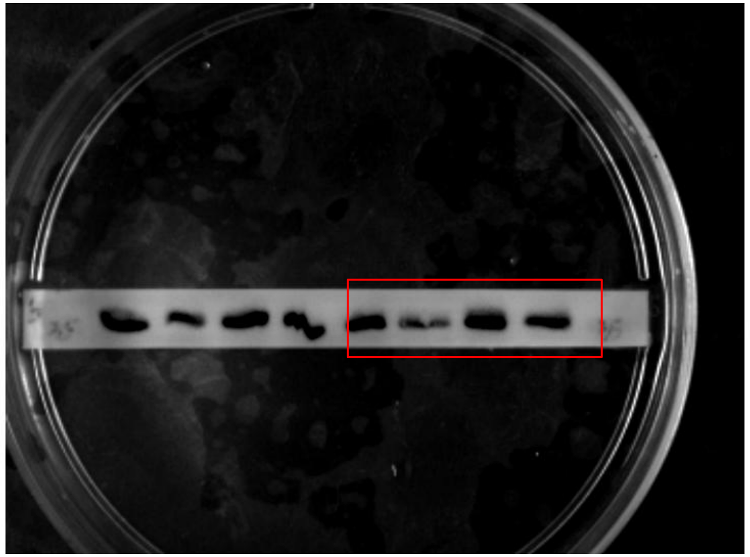

Supplement: Supplementary file 1 [file biology-13-00597-s001.zip › biology-3111520-supplementary.pdf]
