# Supplementary material for: Schisandrin A Attenuates Diabetic Nephropathy via EGFR/AKT/GSK3β Signaling Pathway Based on Network Pharmacology and Experimental Validation
Source: Biology (Basel). 2024 Aug 8;13(8):597. doi: 10.3390/biology13080597 (PMC11351691; doi:10.3390/biology13080597)
Supplement: Supplementary file 1 [file biology-13-00597-s001.zip › CRA Academy WORKSHOPS A Pesic.pdf]

cra akademija

MONITORING MODULE WORKSHOP

Aleksandra Pešić MSc PharmMed CCRA MRQA LLM

Module overview

1. Informed Consent
2. IP Accountability
3. Safety Reporting
4. Selecting Investigators and Sites
5. Investigator Meeting
6. Performing a Site Initiation Visit
7. Clinical Monitoring
8. Site Close Out
9. Audit Process

INFORMED CONSENT

- Discuss purpose, creation and approval of ICDs
- Clarify Informed Consent Process
- Describe special situations requiring extra information and procedures
- Describe the role of ICD in the clinical trial monitoring process
- SAMPLE ICDs

IP ACCOUNTABILITY

Describe and discuss initial IP supply (forecasting, supply requests, verification of shipments)

Describe key procedural issues in IP accountability, including labeling. Blinding, randomization, site monitoring, product recalls, transfer, destruction and donation

Explain the use of Site Monitoring Forms that track and reconcile site inventory with product shipped

Discuss the importance of site IP accountability throughout the clinical trial

IP ACCOUNTABILITY

Case scenario 1:

You arrive at the offices of Austen & Bronte Internal Medicine Associates in Brisbane Australia for a routine monitoring visit. In checking IP shipment records against the Accountability Log you discover 2 boxes of IP required for next month's dispensing are missing from the storage closet

You know that 2 other Pfizer trials are being conducted at this site by 2 other physicians at the practice.

Case scenario 1 discussion:

- What is your first step?
- Assuming that does not rectify the situation what do you do next?
- Is there anything you can do to prevent this from happening again?

CASE SCENARIO 2

- You arrive at the offices of Austen &Bronte Internal Medicine Health Associates in Brisbane Australia 1 month before study close out
- In checking site's supply of IP, you notice a box is missing; in discussion with the site study staff, you learn that the box of product was destroyed by mistake
- Because you know this box of IP is needed for next month's final dispensing you need to act fast; what do you do next?

Case scenario 2 discussion

- Which member of the study team do you consult first?
- What do you ask him or her to do?
- How do you replace the product that was destroyed in error?

CASE SCENARIO 3

- You arrive at the offices of Austen &Bronte Internal Medicine Health Associates in Brisbane Australia 1 month after protocol B2021001 has begun
- In checking entries on dispensing records you notice that 2 subjects only received one bottle of IP (each subject is assigned 2 bottles every 2 months)
- On another form, you see that subjects assigned to comparator drug received an oversupply of 4 extra pills due to a packaging error; there is a note on their charts that each subject was informed of the over-supply by phone the day after dispensing and told to return the unused pills from the incorrectly filled bottle on their next visit, however there is no record that they did return the pills

CASE SCENARIO 3 discussion

- What is the first thing to do?
- How do you get the errors corrected and documented for the file?
- What can you do to prevent this mistake from happening in the future?

SAFETY Inv/CRA responsibilities

- You are monitoring a double-blind, placebo-controlled trial of new daily oral smoking cessation therapy. During the course of the trial, 5 subjects have adverse events that may or may not be related to the study drug.
- Event 1: 2 weeks after starting the investigational therapy , a subject is rushed to the investigator’s hospital after experiencing a myocardial infarction. The subject is currently being treated in intensive care. The physician suspects the study drug has contributed , the investigator agrees..

cra akademija

SAFETY Inv/CRA responsibilities

- Event 2: A subject stubs his toe 2 hours after taking the Investigational Product. The subject telephoned to report the incident; the study nurse took the call and advised the subject to put an ice pack on his toe
- Event 3: A subject reports that she experienced jaundace with oral antibiotics that she had left over from previous prescription. The Investigator believes this may be a recurrence of an infection she had prior to entering the study.

SAFETY Inv/CRA responsibilities

- Event 4: A recently enrolled subject reports severe hearburn lasting several hours each time he takes the study medication. The subject has continued taking the drug, but he asked to withdraw from the trial.The Investigator believes the study drug may be causing the subject's hearburn.
- Event 5: A subject reports that since starting the trial he has experienced erectile dysfunction. On questioning he reveals that he has substituted alcohol for cigarettes and now consistently drinks at least 8 cans of beer per day.

Selecting Investigators

- Identify resources for potential investigators and sites
- Describe the purpose and requirements for a Pre-Study visit
- Explain the steps of the PSV
- Describe the Site Selection

- Investigator Profile: dr. Rose, dr. Johnson, dr. Day
- Pro and cons

INVESTIGATOR MEETING

- Discuss the purpose and timing of Investigator Meetings
- Describe the content of Investigator Meetings and required documentation

PERFORMING SITE INITIATION

- When should the site initiation be conducted?
- Can site initiation occur before the study materials (such as drug) are available at the site?
- If so, what must CRA ensure once the material has arrived?
- Who should attend the Initiation Visit?
- What should the CRA do if the Principal Investigator did not attend the initiation or the Investigator Meeting?

Clinical Monitoring

Define the roles and responsibilities of a CRA

Explain the purpose of monitoring visit

Describe the preparation for monitoring visit

Define remote site monitoring

Explain tasks performed during a monitoring visit

Describe in-office monitoring activities, including the finalization of the report and the submission process

Describe how to handle poor performing or noncompliant sites

Clinical monitoring scenario

- A MV has been arranged for a study on which you are currently working. Your last visit to this site was 1 month ago.
- You leave your office early but due to bad weather your flight is delayed and you arrive at the site at 10:30 am much later than planned 8:30 am for arrival. The PI is only available after 2:00pm and the study nurse has to leave at 4:00pm. Pharmacy is open for you after 1:00 pm. A new part time nurse has started working and works from 09:00am to 12:00 am. 10 patients enrolled, 7 randomized. The PI is leaving for 3 weeks vacation tomorrow.

SITE CLOSE OUT

- You have arrived at the site to conduct your end of study visit. During your visit, you become aware of the following issues:
- The study coordinator tells you that 3 subjects completed their final visit since your last monitoring visit; when performing CRF review and source document verification for these final visits you discover that the corresponding lab reports for these subjects are missing
- The protocol and the IRB/IEC approval letter are not in the site file
- The Investigator tells you that he is moving practice to a new location at the beginning of the year
